# Single cell and lineage tracing studies reveal the impact of CD34^+^ cells on myocardial fibrosis during heart failure

**DOI:** 10.1186/s13287-023-03256-0

**Published:** 2023-02-20

**Authors:** Luping Du, Xiaotong Sun, Hui Gong, Ting Wang, Liujun Jiang, Chengchen Huang, Xiaodong Xu, Zhoubin Li, Hongfei Xu, Liang Ma, Weidong Li, Ting Chen, Qingbo Xu

**Affiliations:** 1 Department of Cardiology, School of Medicine, The First Affiliated Hospital, Zhejiang University, 79 Qingchun Road, Hangzhou, 310003 Zhejiang China; 2 Department of Lung Transplantation, First Affiliated Hospital, School of Medicine, Zhejiang University, Hangzhou, 310003 China; 3 Department of Cardiovascular Surgery, First Affiliated Hospital, School of Medicine, Zhejiang University, 79 Qingchun Road, Hangzhou, 310003 Zhejiang China; 4 Alibaba-Zhejiang University Joint Research Center of Future Digital Healthcare, Hangzhou, China

**Keywords:** CD34^+^ cells, Single-cell RNA sequencing, Lineage tracing, Myocardial fibrosis, Heart failure

## Abstract

**Background:**

CD34^+^ cells have been used to treat the patients with heart failure, but the outcome is variable. It is of great significance to scrutinize the fate and the mechanism of CD34^+^ cell differentiation in vivo during heart failure and explore its intervention strategy.

**Methods:**

We performed single-cell RNA sequencing (scRNA-seq) of the total non-cardiomyocytes and enriched Cd34-tdTomato^+^ lineage cells in the murine (male Cd34-CreERT2; Rosa26-tdTomato mice) pressure overload model (transverse aortic constriction, TAC), and total non-cardiomyocytes from human adult hearts. Then, in order to determine the origin of CD34^+^ cell that plays a role in myocardial fibrosis, bone marrow transplantation model was performed. Furthermore, to further clarify the role of CD34 + cells in myocardial remodeling in response to TAC injury, we generated Cd34-CreERT2; Rosa26-eGFP-DTA (Cre/DTA) mice.

**Results:**

By analyzing the transcriptomes of 59,505 single cells from the mouse heart and 22,537 single cells from the human heart, we illustrated the dynamics of cell landscape during the progression of heart hypertrophy, including CD34^+^ cells, fibroblasts, endothelial and immune cells. By combining genetic lineage tracing and bone marrow transplantation models, we demonstrated that non-bone-marrow-derived CD34^+^ cells give rise to fibroblasts and endothelial cells, while bone-marrow-derived CD34^+^ cell turned into immune cells only in response to pressure overload. Interestingly, partial depletion of CD34^+^ cells alleviated the severity of myocardial fibrosis with a significant improvement of cardiac function in Cd34-CreERT2; Rosa26-eGFP-DTA model. Similar changes of non-cardiomyocyte composition and cellular heterogeneity of heart failure were also observed in human patient with heart failure. Furthermore, immunostaining showed a double labeling of CD34 and fibroblast markers in human heart tissue. Mechanistically, our single-cell pseudotime analysis of scRNA-seq data and in vitro cell culture study revealed that Wnt-β-catenin and TGFβ1/Smad pathways are critical in regulating CD34^+^ cell differentiation toward fibroblasts.

**Conclusions:**

Our study provides a cellular landscape of CD34^+^ cell-derived cells in the hypertrophy heart of human and animal models, indicating that non-bone-marrow-derived CD34^+^ cells differentiating into fibroblasts largely account for cardiac fibrosis. These findings may provide novel insights for the pathogenesis of cardiac fibrosis and have further potential therapeutic implications for the heart failure.

**Supplementary Information:**

The online version contains supplementary material available at 10.1186/s13287-023-03256-0.

## Introduction

Heart failure represents a leading cause of morbidity and mortality worldwide, in which pathological ventricular hypertrophy is an independent risk factor [1]. There are two events related to the pathogenesis of heart failure—endothelial dysfunction and fibrosis [2, 3]. The former is involving in increased expression of pro-inflammatory genes/proteins and angiogenesis [4], while the fibrosis is a consequence of fibroblast-produced matrix protein deposition that contributes to systolic and diastolic dysfunction and the altered electrical signal transduction [5]. Fibroblasts are the dominant non-cardiomyocytes and the main effector cells of myocardial fibrosis and heart failure [6, 7]. There are various reports on the changes of fibroblast subsets [8], e.g., quiescent, activated, myofibroblast and matri-fibrocytes. The heterogeneity and subtypes of fibroblasts could exert their effect on myocardial fibrosis in different ways, to which a variety of sources of cells may contribute. However, it remains unclear whether different subtypes of fibroblasts can be derived from mature fibroblasts or stem/progenitor cells in the development of cardiac fibrosis.

Over the past decade, it is well known that resident cells that express c-Kit, Sca-1 and CD34 cannot differentiate into cardiomyocytes [9–12], but they do have an ability to differentiate into endothelial cells and other non-cardiomyocytes in response to myocardial injury [13]. For instance, CD34^+^ cells exist in tissues, circulating blood and bone marrow, which may serve as a source of different types of cells in pathological status [14]. Bone marrow-derived CD34^+^ cell therapy has received great attention for its potential applications in cardiovascular disease [15–17]. Several studies have shown that injection of CD34^+^ cells into the heart is safe and might be effective to reduce the progression of cardiac remodeling after ischemic injury [18–21]. However, the results obtained from clinical trials using these cells have been inconsistent [18, 22–24]. The explanation for the diverse outcome of the CD34^+^ cell therapy is due to lacking the information about basic biological nature of CD34^+^ cells in the tissue and blood, e.g., the function and fate of CD34^+^ cells in the development of cardiac remodeling.

Herein, we used *Cd34*-CreER;Rosa26-tdTomato mice, an inducible lineage tracing model, to trace the fate of CD34^+^ cells in TAC (transverse aortic constriction)-induced myocardial remodeling, then combined with single-cell RNA sequencing (scRNA-seq) technique and bone marrow transplantation model; our results showed that non-bone-marrow-derived CD34^+^ cells are the major source of fibroblasts and endothelial cells in the process of myocardial fibrosis. Furthermore, our study provided the solid evidence that ablation of CD34^+^ cells can alleviate the myocardial fibrosis. Mechanistically, we confirmed the fibroblastic differentiation potential of isolated CD34^+^ cell in vitro involving in Wnt-β-catenin and TGFβ1/Smad pathways.

## Materials and methods

### Mice

All animal experiments and protocols were performed in accordance with the National Institutes of Health’s Guide for the Care and Use of Laboratory. Animals were approved by the Research Ethics Committees of the First Affiliated Hospital of Zhejiang University. In this study, we used the following mouse strains: CD34-CreERT2 knock-in mice (C57BL/6 background) and Rosa26-tdTomato mice (B6.Cg-Gt(ROSA)26Sor^tm9(CAG−tdTomato)Hze^/J)(JAX: 007,909) were purchased from Shanghai Biomodel Organism Co., Ltd. Rosa26- eGFP-DTA (JAX: 006,331) mice were purchased from the Jackson Laboratory, USA. CD34-CreERT2; Rosa26-tdTomato and CD34-CreERT2; Rosa26-tdTomato -DTA mice were constructed as described in the previous paper [25].

Mice were maintained at temperature (22 ± 1 °C) and humidity (65–70%) controlled room, with a 12-h light and 12-h dark cycle and allowed free access to chow and water. All mice generated or purchased were housed for at least one week before use.

### Human heart samples

In this study, we included two types of human left ventricular samples: one control sample (non-failing, non-transplantable hearts) and one heart failure sample (with a history of hypertension). The control heart was collected from a healthy organ donor, which was deceased due to acute trauma, and the heart had the EF of greater than 50%, and was considered to be not suitable for transplantation. The left ventricles of heart failure were collected from patients undergoing heart transplantation. The heart failure patient was diagnosed with EF < 30% (systolic heart failure).

### Transaortic aorta constriction (TAC) model

Transverse aortic constriction surgery was performed as described previously [26]. For analysis, adult male mice (n = 100) weighing 25 ± 5 g were randomly allocated to different groups (TAC 0, 4 and 12 week(s) post-operation groups). The numbers of mice were indicated in the figure legends for each experiment.

As for the bone marrow transplantation (BMT) experiments, there were two groups of animal experiments. (Chimeric mice were created by transplanting bone marrow cells from wild-type C57BL/6 J mice to CD34-CreERT2;Rosa26-tdTomato, or from CD34-CreERT2;Rosa26-tdTomato to wild-type C57BL/6 J mice, further subjected to TAC 4-week surgery.)

As for the Cd34 + cell depletion experiments, we performed 4-week TAC surgery on CD34-CreERT2; Rosa26-tdTomato -DTA mice and CD34-CreERT2;Rosa26-tdTomato.

At indicated timepoints after TAC surgery, mice were euthanized using carbon dioxide (CO_2_) with minimal stress to them. Briefly, a cage that containing 5 mice was placed in a separate 20-L volume chamber and performed as described previously [25]. The mice were then removed from the cage, and the heart tissues were harvested. After TAC, mice were only excluded/ euthanized humanely in time when the animal health condition was poor.

### Echocardiography

Mouse heart function was assessed by transthoracic ultrasonography imaging with Vevo 770 high-resolution ECHO system [27]. All mice underwent echocardiography 0, 4 and 12 weeks after TAC surgery.

### CD34^+^ cell depletion experiment

To remove the CD34^+^ cells in mice, tamoxifen was administered to the *Cd34*-CreER; Rosa26-tdTomato-DTA mice by gavage over two weeks. Cardiac function is determined by echocardiography and performed four weeks after the transverse aortic constriction (TAC) experiment.

### Histology and trichrome staining

Hematoxylin and eosin (H&E), Masson’s trichrome staining and Picro-Sirius red staining (400 × for Histopathological staining studies) were performed according to manufacturer’s instructions. And we used the slide scanner (3DHISTECH, PANNORAMIC 250) to acquire images.

### Immunofluorescence staining

Heart tissue immunofluorescence staining was performed as described previously [25], the Nikon A1R confocal laser scanning microscope (Nikon, Tokyo, Japan) was used to acquire cryo-section staining images (the type of equipment: Ti microscope; Optics, Plan Apo VC 20X DIC N2; Camera, Nikon A1 plus; Numerical aperture, 0.75; Scanner selection, Galvano; Detector selection, DU4; Filter model, filter cube, 450/50, 525/50, 595/50;), and the measured resolution of the images was 2048 X 2048 at the line average model. Regions were selected randomly to avoid biasing. As for quantification of immunostaining images, we used Image J to analyze the images from 3–5 different sections.

Primary antibodies were Vimentin (1:200, abcam, ab8978), DDR2 (1:200, R&D, MAB25381), PDGFRA (1:100, R&D, AF1062), POSTN (1:100, R&D, AF2955), THBS4 (1:100, R&D, AF2390), CD34 (1:100, abcam, ab81289), CD31 (1:100, R&D, AF3628), RFP antibody (1:300, Rockland, 600–401-379). Alexa Fluor-conjugated secondary antibodies used in this study included Donkey anti-Mouse IgG (1:500, Invitrogen, A21202 for Alexa Fluor 488), Donkey anti-Goat IgG (1:500, Invitrogen, A11055 for Alexa Fluor 488, A21447 for Alexa Fluor 647), Donkey anti-Rabbit IgG (1:500, Invitrogen, A31572 for Alexa Fluor 555).

### FACS analysis

Isolation of cells from the freshly collected cardiac tissue was performed by cutting the heart into pieces, following by digestion buffer (Liberase, Roche) at 37 °C for 15 min. All prepared cells were suspended in PBS and then conjugated with antibodies for 30 min where indicated. Antibodies used in this study include LIVE/DEAD Fixable Near-IR Dead Cell Stain Kit (1:1000, Invitrogen, L34975), Hoechst (1:1000, Invitrogen, H3570), PDGFRA-APC (1:100, Invitrogen, 17–1401), CD31-APC (1:100, Invitrogen, 17–0319-42), CD34-FITC (1:100, BD Biosciences, 560,238). Cells were then analyzed by using the BD FACSVerse Flow Cytometer (BD Biosciences).

### Bone marrow transplantation (BMT)

This transplantation experiment was performed as previously described [28]. The bone marrow cells were resuspended in RPMI 1640 before transplantation, and the irradiated recipient mice (A lethal dose of whole-body irradiation (9.0–9.5 Gy)) received 5 × 10^6^ bone marrow cells from donor to form chimeric mice via tail vein injection six hours later.

### Single-cell dissociation and cell sorting

To obtain single cells from the human or mouse heart, a similar protocol was followed as previously described [29] after collecting the heart of TAC 0 or TAC-operated mice or human hearts.

Then, the cells resuspended in PBS after lysing red blood cells and filtering with 40-μm filter. After staining with Hoechst (1:1000, Invitrogen, H3570) and Dead Cell Stain Kit (1:1000, Invitrogen, L34975), nucleated live cells were sorted into PBS with 0.04% BSA for subsequent scRNA-seq by using the BD FACS ARIA II Flow Cytometer.

### Processing of scRNA-seq data

We performed scRNA-seq of six pooled samples from 0, 4 and 12 weeks after TAC surgery separately. For scRNA-seq data analyses of the human or mouse heart, a similar protocol was followed [25]. Raw scRNA-seq data were processed using the 10 × Genomics Cell Ranger software (version 3.1.0). Analyses of scRNA-seq data were carried out in R version 4.0 by using the Seurat suite versions 3.2.3. As for the initial quality control filtering, we excluded the low-quality cells (< 400 genes/ cell, > 20,000 genes/ cell and > 10% mitochondrial transcript presence/ cell). Then, we used the Seurat v3 with default parameters to integrate the datasets collected from different samples and remove the batch effect.

Dimensionality reduction using PCA was undertaken to explore the heterogeneity. UMAP was used for reduction to two dimensions for visualization purposes. Markers for a specific cluster were found with function FindAllMarkers (min.pct = 0.25, logfc.threshold = 0.25).

### Mouse heart CD34^+^ cell isolation and cell culture

Isolation of mouse heart cells and CD34^+^ cells was performed as previously described [29, 30]. Then, cells were sorted with anti-CD34 magnetic beads (Miltenyi Biotec) according to manufacturer’s instructions. The cells were cultured as specified previously [31]. The purified CD34^+^ cells were seeded onto dishes with 0.1% gelatin (Sigma, G1393) and maintained in complete stem cell culture medium, which consists of DMEM, 1% FBS, 2% chick embryo extract (MP Biomedical), 100 nM retinoic acid (Sigma-Aldrich), 50 nM 2-mercaptoethanol (Sigma-Aldrich), 2% B27 (Invitrogen), 1% N2 (Invitrogen), 20 ng /ml bFGF (R&D Systems) and 1% P/S.

For cell differentiation experiments, CD34 + cells were cultured on gelatin-coated plates and maintained in complete stem cell culture with 50 ng/mL CTGF (Recombinant Human Connective Tissue Growth Factor, PeproTech, 120–19-20), which is sufficient to differentiate MSCs into fibroblast cells [32]. In the presence of recombinant TGFβ1 (5 ng/mL) (R&D systems, 7666-MB-005), cells were also treated with inhibitors including SB525334 (a selective TGFβ1 receptor inhibitor) (2 μM).

### RT-PCR analysis

Total RNA was extracted with TRIzol Reagent and converted into cDNA by using the cDNA Reverse Transcription Kit (Promega, Madison, USA). And the relative quantification of mRNA was analyzed by using the CFX96 Real-Time System (BIO-RAD, CA, USA).

The primers used were as follows:

- Col1: forward: 5’- GCCAAGAAGACATCCCTGAAG-3’; reverse: 5’- TGTGGCAGATACAGATCAAGC-3’); Col1a2: forward: 5’- GCCACCATTGATAGTCTCTCC-3’
- reverse: 5’- CACCCCAGCGAAGAACTCATA-3’); Col3a1: forward: 5’- TCCCCTGGAATCTGTGAATC-3’
- reverse: 5’- TGAGTCGAATTGGGGAGAAT-3’); Vimentin: forward: 5’- CGGCTGCGAGAGAAATTGC-3’
- reverse: 5’- CCACTTTCCGTTCAAGGTCAAG-3’); Ddr2: forward: 5’- CTGTGGGAGACCTTCACCTT-3’
- reverse: 5’- TAGATCTGCCTCCCTTGGTC-3’); PDGFR-α: forward: 5’- GGACTTACCCTGGAGAAGTGAGAA-3’
- reverse: 5’- ACACCAGTTTGATGGATGGGA-3’); Fn1: forward: 5’- AAGGCTGGATGATGGTGGAC-3’
- reverse: 5’- TGAAGCAGGTTTCCTCGGTTG-3’); Gapdh: forward: 5’- TGTCGTGGAGTCTACTGGTG-3’
- reverse: 5’- ACACCCATCACAAACATGG-3’); α-SMA: forward: 5’- GAGAAGAGCTACGAACTGCC-3’
- reverse: 5’- CATCCTGTCAGCAATGCCTG-3’); Periostin: forward: 5’- ACGGAGCTCAGGGCTGAAGATG-3’
- reverse: 5’- GTTTGGGCCCTGATCCCGAC-3’); WNT5A: forward: 5’- GGTGGTCGCTAGGTATGAATAA-3’
- reverse: 5’- TCTTCTGTCCTTGAGAAAGTCC-3’);

### Western blot analysis

Cells were lysed in RIPA lysis buffer supplemented with phosphatase inhibitor tablets (Roche, 04,906,837,001) and protease inhibitor tablet (Roche, 05,892,970,001). Primary antibodies were Vimentin (Cell Signaling, 5741,1:1000), DDR2 (R&D, MAB25381, 1:2000), PDGFRA (R&D, AF1062, 1:1000), Periostin (R&D, AF2955, 1:1000), Wnt5a (Cell Signaling, 2530, 1:1000), β-Actin (Cell Signaling, 4970, 1:2000), β-Catenin (Cell Signaling, 8480, 1:2000), α-SMA (Sigma, A5228, 1:2000), Fibronectin (Cell Signaling, 26,836, 1:1000), Collagen I (abcam, ab6308, 1:1000), GAPDH (Cell Signaling, 5174, 1:2000), Smad2 (Cell Signaling, 5339, 1:1000), Phospho- Smad2 (Cell Signaling, 3108, 1:1000).

### Mouse serum ELISA experiment

The levels of ANP and BNP in mouse serum were detected by Mouse ANP ELISA kit (Abcam, ab267800) and Mouse BNP ELISA kit (Novus, NBP2-70,011) according to the protocol provided.

### siRNA transfection

Cells with > 80% sub-confluency were transfected with β-Catenin siRNA using Lipofectamine RNAiMAX (Invitrogen). After transfection for 48 h, relevant experiments were carried out.

### Gene ontology analysis

We used the FindAllMarkers function to perform the gene ontology (GO) and genomes pathway analysis of each cluster or through the enriched genes found by FindMarkers function with average log (fold change) > 0.25 on Metascape website.

### Gene sets and cell cycle analysis

The Seurat function “AddModuleScore” was used to perform gene set analyses, including endothelial subtype markers and immune cell type genes obtained from previous published studies and PanglaoDB website [33, 34]. And the “CellCycleScoring” of seurat function was used to assess the state of cell cycle, using a list of cell cycle gene sets from a previous study [35].

### Pseudotime trajectory analysis

The pseudotime analysis was perform by Monocle (2.16.0). Briefly, we reduced the dimensionality of our datasets using “DDRTree” method, and ordered cells along the pseudotime trajectory using the differentially expressed genes acquired in the Seurat analysis.

### Ligand–receptor cellular communication network analysis

CellPhoneDB [36] was used to predict the specificity of cell–cell communications and also identified the locations of ligand–receptor interactions among single cells.

### Statistical analysis

Investigators measuring Western blotting intensity were blinded with respect to the experimental protocols. GraphPad Prism 9.0 was used to draw statistical images and perform comparative analyses. The data were presented as mean ± SEM. Prior to analysis, all data sets were tested for normality distribution by Shapiro–Wilk test, followed by using the Student's t-test or one-way ANOVA followed by Tukey's test. *P* < 0.05 was considered to be statistically significant.

## Results

### scRNA-seq analysis of cardiac hypertrophy in time course

**Fig. 1:**
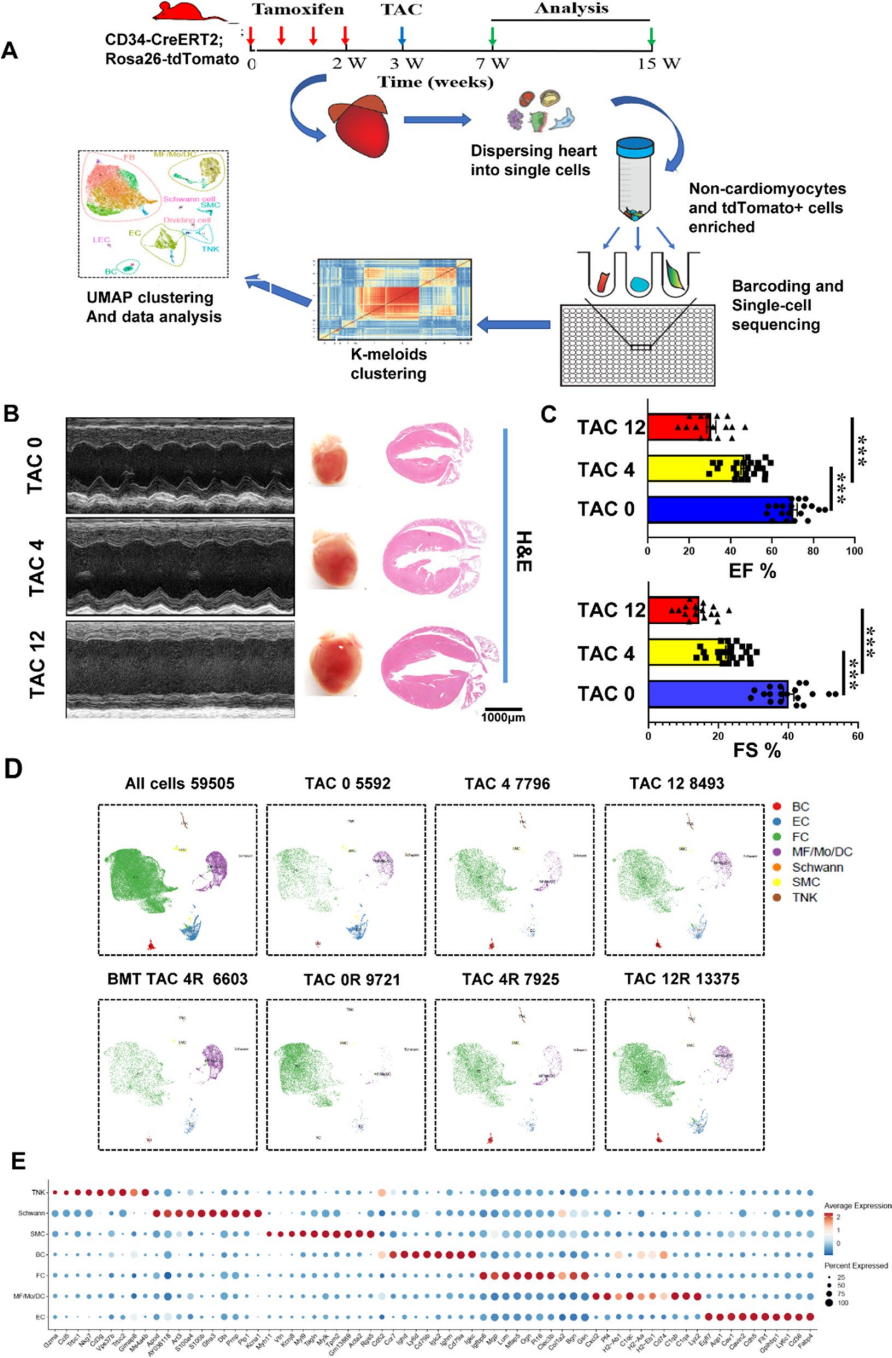
Single-cell RNA sequencing (scRNA-seq) analysis of cardiac hypertrophy in a mouse model of pressure overload. **A** Schematic depicting the pipeline of transverse aortic constriction (TAC) surgery and tissue harvesting of *Cd34*-CreERT2; Rosa26-tdTomato mouse for scRNA-seq. Heart tissue was harvested 0, 4 and 12 weeks after TAC surgery. Single cells were then isolated from the left ventricle and subjected to scRNA-seq. N = 6 per group. **B** Representative echocardiography of mice at different stages of pathological cardiac hypertrophy; H&E staining of heart at 0, 4 and 12 weeks after TAC surgery. **C** Echocardiographic measurements of left ventricle ejection fraction (EF) and fractional shortening (FS) in mice from TAC 0 or different TAC groups. TAC 0 group, n = 19; TAC 4 group, n = 23; TAC 12 group, n = 16. Data represent mean ± SEM. *P < 0.05; **P < 0.01; ***P < 0.001. **D** Umap plot displaying the major cell types and color-coded cell clusters at different stages of pathological cardiac hypertrophy. n = 59,505 cells. **E** Dot plot showing expression levels of top five differentially expressed genes in each cell cluster. Dot size reflects the percentage of cells expressing the selected gene in each cell cluster

To understand the pathological procession during the heart failure, a model of transverse aortic constriction (TAC)-induced pressure overload at different phases, including the control (TAC 0), hypertrophy with reduced ejection fraction (TAC 4: 4 weeks, early heart failure), and heart failure (TAC 12: 12 weeks, terminal heart failure), was established. TAC operation led to a significant decrease of cardiac functionality in comparison with TAC 0. However, the left ventricular ejection fraction (LVEF) and fractional shortening (FS) were the lowest in the heart failure phase (Fig. 1B, C). Cardiac tissues were collected after TAC injury, H&E, Masson and Sirius red staining on cardiac sections also indicated that myocardial fibrosis dramatically increased after the TAC surgery, whereas no fibrosis area was observed in the TAC 0 group (Fig. 1B; Additional file 1: Figure S1A).

Up to now, growing evidence shows that non-cardiomyocytes play crucial roles in heart development and diseases [26, 37, 38]. Thus, we aimed to delineate the dynamic alterations of major non-cardiomyocyte cell types during pathological cardiac hypertrophy. Single nucleated live cells were isolated from hearts of 8–10-week-old male mice, 4 and 12 weeks after TAC operation, by enzymatic digestion and sorting the cardiac non-cardiomyocyte cells, further processed for scRNA-seq using the 10 × genomics chromium platform (Fig. 1A; Additional file 1: Figure S1B). We systematically investigated the pathological progression of cardiac hypertrophy by sequencing 69,964 single cells, from left ventricle regions of mouse hearts at representative stages (0, 4 and 12 weeks) after TAC-induced pressure overload, a total of 59,505 cells were captured after quality control filtering (Additional file 1: Figure S1C). Following quality control, to identify cells with distinct lineages and transcriptional states, we performed unbiased clustering on an aggregate of cells using the Seurat [39], and cell populations were visualized in UMAP dimensionality reduction plots [40]. A total of 7 major cell types were identified including fibroblasts (FB; *Dcn* + *Ddr2* +), endothelial cell (EC; *Cdh5* + *Kdr* +), T cells and natural killer cell (*Cd3g* + *Nkg7* +), macrophage/monocyte/dendritic cell (MF/Mo/DC; *Adgre1* + *Csf1r* +), B cell (BC; *Cd79a* + *Cd19* +), smooth muscle cell (SMC; *Acta2* + *Tagln* +) and Schwann cell (*Plp1* + *Nrn1* +), based on their respective molecular signatures (Fig. 1D, E; Additional file 1: Figure S1D).

### scRNA-seq analysis of non-cardiomyocytes at different stages of cardiac hypertrophy

**Fig. 2:**
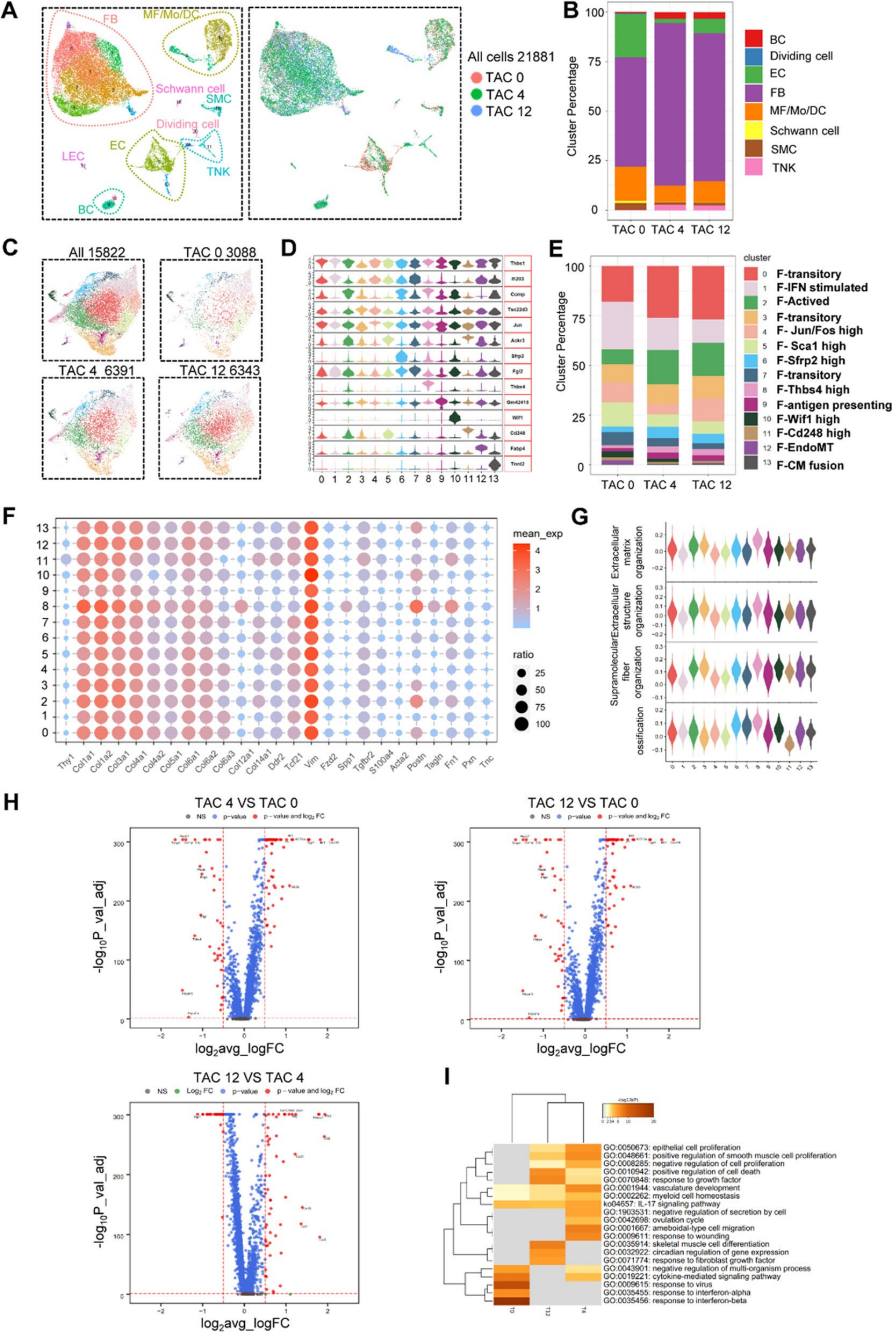
Characterization of heart composition at different stages of pathological cardiac hypertrophy by ScRNA-seq. **A** Umap plot displaying the major cell types and color-coded cell clusters of CD34-CreERT2; Rosa26-tdTomato mouse at 0, 4 and 12 weeks after TAC surgery. n = 21,881 cells. **B** Bar chart showing the percentage of major cell types among different datasets at different stages of pathological cardiac hypertrophy. **C**. Umap plot displaying distribution of fibroblast subpopulations among different datasets at 0, 4 and 12 weeks after TAC surgery. n = 15,822 cells. resolution = 0.5. **D** Violin plot showing the expression of selected marker gene of each subclusters. **E** Bar chart showing the percentage of subclusters in datasets at 0, 4 and 12 weeks after TAC surgery. **F** Dot plot showing the expression of fibroblast genes. Dot size reflects the percentage of cells expressing the selected gene in each cell cluster. **G** Violin plot showing the GO datasets score among fibroblast subclusters. **H** Volcano plot showing the DEGs between datasets (p-value < 0.01 and log2FC > 1 was labeled). **I** Go analysis revealed biological processes enriched with DEGs between different groups (0, 4 and 12 weeks after TAC surgery) (DEGs log2FC > 0.5 and pct.1/pct.2 > 1 were used). T indicates TAC

To determine cellular landscape of cardiac non-cardiomyocytes in response to the TAC injury, we obtained single-cell transcriptomes for 21,881 cells by integrating three groups from our mouse models (TAC 0, TAC 4, TAC 12) after strict quality control (Additional file 1: Figure S1C) (Fig. 2A). Examination of cell abundances within this scRNA-seq data set revealed several cell populations that changed in relative prevalence in response to TAC surgery. In line with previous reports, we observed the proportion of fibroblast was significantly higher in TAC groups than the control group (Fig. 2B).

Given this situation and the vital role of fibroblasts in fibrosis [41], these cells were partitioned into distinct subtypes comprising cells from all the groups. The dispersion of fibroblast clusters in the UMAP plots indicated a high degree of fibroblast heterogeneity (Fig. 2C). Notably, several distinct fibroblast subtypes show obvious changes in constitution percentage in the TAC groups, compared with the control groups (Fig. 2D). In our datasets, we identified several fibroblast subclusters reported in previous scRNA-seq [42] study of cardiac hypertrophy induced with Angiotensin II, including fibroblast-*Thbs4* and fibroblast-*Wif1* (Fig. 2D). Consistent with previous reports [42], activation of fibroblast was evidenced by increased proportions of cluster 2, cluster 6 and cluster 8 (subtypes with high expression of extracellular matrix protein including *Comp*, *Postn*, *Thbs4*) at the stage of cardiac hypertrophy (Fig. 2E, F). Fibroblasts mentioned above show elevated capacity of ECM organization, extracellular structure organization, supramolecular fiber organization and ossification, which are involved in fibrosis development (Fig. 2G). Cluster 10 (fibroblast-*Wif1*) showed increased expression of genes (*Wif1*, *Dkk3*, *Sfrp1*, *Sfrp2*) involved in negative regulation of Wnt signaling pathway, which has been considered to inhibit cardiac fibrosis (Figure S2A-B). Considered that fibroblast-*Wif1* played a negative role in cardiac fibrosis development, the decreasing percentage of fibroblast-*Wif1* during the progress of cardiac hypertrophy may be a reason for heart function deterioration. Since cluster 12 enriched the expression of genes related to EC function, we defined it as a subcluster undergoing endothelial to mesenchymal transition (EndoMT) (Additional file 1: Figure S2A-B). Furthermore, we noticed that cluster 12 expressing cardiomyocytes marker genes (e.g., *Ankrd1*, *Mb*, *Tnnt2*), there are some possible explanations for this case: (1) it has been reported that some non-cardiomyocytes may express cardiomyocytes genes. (2) cluster 12 may be the ‘hybrid’ cells population, which contains doublets in proximity due to uncomplete dissociation. (3) cluster 12 may be a cluster-presenting cell fusion.

**Fig. 3:**
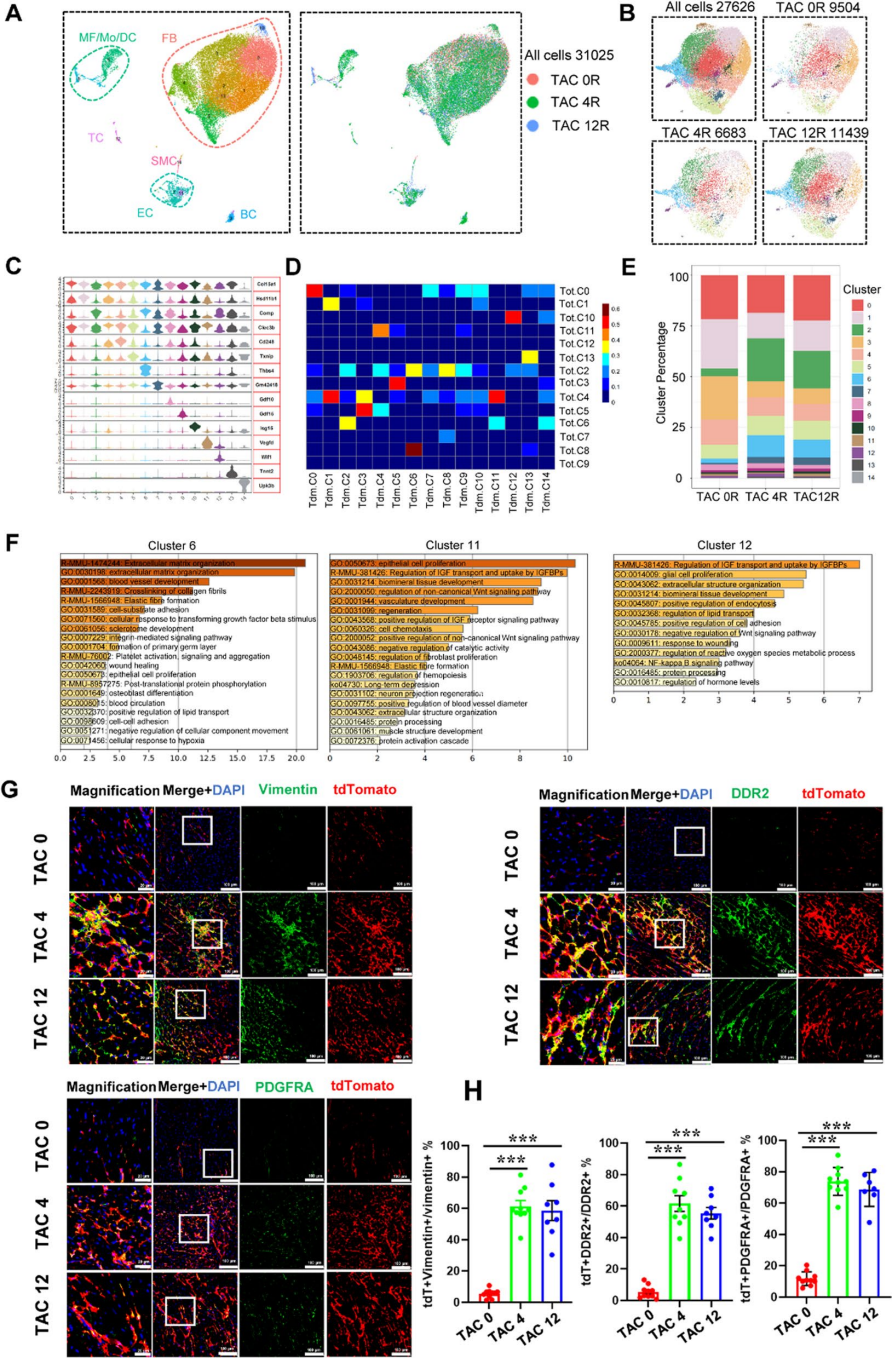
Characterization of CD34 cell-derived cells at different stages of pathological cardiac hypertrophy by ScRNA-seq.** A** Umap plot displaying the major cell types and color-coded CD34 derived cell clusters of Cd34-CreERT2; Rosa26-tdTomato mouse at 0, 4 and 12 weeks after TAC surgery. n = 31,025 cells. **B** Umap plot displaying distribution of fibroblast subpopulations among different datasets at 0, 4 and 12 weeks after TAC surgery. n = 27,626 cells. resolution = 0.35. **C** Violin plot showing the expression of selected marker gene of each subcluster. **D** Heatmap showing cluster similarity of fibroblast subclusters in total and CD34 + cell-derived datasets. **E** Bar chart showing the percentage of subclusters in datasets at different stages of pathological cardiac hypertrophy. **F** Bar plot showing the GO enrichment of selected subclusters. **G** Representative images showing specific cell identification by staining with tdTomato, Vimentin, DDR2, PDGFRA (Scale bar = 20 μm and 100 μm). n = 8 per group. **H** Graph showing percentage of tdTomato expression in vimentin + , DDR2 + , PDGFRA + fibroblast. Data represent mean ± SEM, n = 8. *P < 0.05; **P < 0.01; ***P < 0.001. Tdm, tdTomato

Fibroblasts play a critical role in cardiac remodeling by participating in ECM generation and organization. Up-regulated differentially expressed genes (DEGs) of different datasets showed that compared with TAC 0 group, the TAC groups significantly enriched genes involved in cardiac fibrosis, including *Ctgf*, *Postn*, *Comp* and *Thbs4*. And compared with TAC 12, TAC 4 datasets showed elevated expression of multiple chemokine genes, suggesting that short period of TAC was more abundant in recruiting inflammatory cells (Fig. 2H). GO analysis of these datasets demonstrated that the TAC group significantly enriched several biological processes related to cardiac remodeling (Fig. 2I). Consistently, *Vimentin*, *Postn*, *Ddr*2 and *PDGFRa* expression were increased in heart sections during disease progression (Fig. 3G, H; Additional file 1: Figure S3F), and the percentage of *PDGFRa*^+^ cell in the heart was also increased by flow cytometry analysis (Additional file 1: Figure S2H).

Further GO enrichment analysis showed that extracellular structure organization, cardiac muscle tissue growth, blood vessel development and vasculogenesis were highly enriched in *Cd34*^high^ fibroblasts compared with the *Cd34*^low^ fibroblasts (cluster 11 (Additional file 1: Figure S2C), in addition, cluster 11 showed elevated expression of multiple progenitor cell markers, including *Ly6a, Klf4* (Additional file 1: Figure S2D), all these indicating that *Cd34*^high^ fibroblasts may exhibit specific functions during cardiac homeostasis and after injuries.

Thus, these led us to investigate the potential for a developmental relationship among these subclusters of fibroblasts. Then, the pseudotime(s) visualized using principal curves represent trajectories of fibroblast differentiation across steady-state atlas with cluster 11 set as root. The fibroblasts were ordered along a trajectory, and cells at different states were identified (Additional file 1: Figure S2E-F). Furthermore, the expression of *Cd34* and *Ly6a* significantly changed with the states, in correspondence of pseudotime trajectory (Additional file 1: Figure S2G). Furthermore, the CD34 lineage fibroblast markers (including *Vimentin*, *Postn*, *Ddr*2 and *PDGFRa*) were increased in heart tissues during disease progression by immunofluorescence staining (Fig. 3G, H; Additional file 1: Figure S3F and Figure S4E).

Combined with these all data, we identified CD34^+^ cells, at least a subpopulation in the heart, has a progenitor cell-like phenotype, which could transdifferentiate into multiple cell types especially the fibroblasts, and plays the specific functions in cardiac fibrosis.

### Lineage tracing study reveals CD34^+^ cells at different stages by scRNA-Seq

In order to investigate the specific functions of CD34^+^ cells during cardiac fibrosis after injuries, we next used an inducible genetic lineage tracing mouse model, *Cd34*-CreER^T2^;Rosa26-tdTomato knock in mouse (Additional file 1: Figure S3A). Mice were treated with five consecutive pulses of tamoxifen for 1 week to induce tdTomato labeling of CD34^+^ cells, and heart tissues were collected for analysis 1 week later (Additional file 1: Figure S3A). To verify successful induction of tamoxifen, *Cd34*-CreERT2; Rosa26-tdTomato mice with or without tamoxifen induction were performed with in vivo imaging system (Additional file 1: Figure S3B). Whole-mount staining of the heart of the two groups above also confirmed successful induction of Cre-recombinase (Additional file 1: Figure S3C). Immunofluorescence staining was applied to verify the specificity and sensitivity of *Cd34*-CreERT2;Rosa26-tdTomato mice (Additional file 1: Figure S3D). Echocardiograph imaging was also performed to evaluate cardiac function of the two different genotypes mice, respectively, including left ventricle ejection fraction (LVEF) and FS, which showed no significant difference in cardiac function between the *Cd34*-CreER^T2^; Rosa26-tdTomato and wild-type animals (Additional file 1: Figure S3E).

To delineate the constitution map of cardiac resident CD34 lineage cells during different stages of cardiac hypertrophy, we performed scRNA-seq in CD34 lineage cells (tdTomato^+^ cells). Total number of 31,045 cells was available for further analysis after strict quality control (Fig. 3A). Based on the transcriptome characteristics, cells were divided into six major cell types including fibroblast, EC, SMC, B cells, NK cells and macrophages (Fig. 3A). We focused on the CD34 lineage fibroblast to discover their heterogeneous features among the three group (TAC 0, TAC 4, TAC12) (Fig. 3B). The extracted fibroblast population was further clustered into 15 subclusters. Several fibroblast subclusters, including *Cd248*, *Thbs4* and *Wif1*, showed significant similarity between CD34 lineage fibroblasts populations and original datasets (Fig. 3C, D). We also found some new fibroblast subclusters differed from those in the original datasets. Cluster 14 (*Upk3b*) was a specific cluster expressing mesothelial cells markers, suggesting a minor role of transition between mesothelial cell and fibroblasts in cardiac hypertrophy development (Fig. 3C; Additional file 1: Figure S4A). Cluster 11 (*Vegfd*) was a newly identified subcluster highly expressing *Vegfd* and *Wnt* pathway-related genes. GO analysis revealed that cluster11 enriched regulation of non-canonical *Wnt* signaling pathway, vasculature development and regulation of fibroblast proliferation (Fig. 3F). The percentage of cluster 11 in CD34 lineage fibroblast population decreased in 4-week period but increased in 12 weeks (Fig. 3E). Compared with TAC 0 group, the percentage of multiple fibroblast clusters (including clusters 2, 5, 6 and 7) were significantly higher in the TAC group (Fig. 3E). Several genes associated with ECM proteins and fibroblast activation were increased in the hypertrophy group compared to the TAC 0 group (Additional file 1: Figure S4B-D). GO analysis of DEGs of different groups revealed that hypertrophy group significantly enriched several biological processes which involving in cardiac remodeling, vasculature development, ossification and elastic fiber formation (Figure S4C). Consistently, the significantly increase of CD34 lineage fibroblast was also identified by immunofluorescence staining (Fig. 3G, H; Additional file 1: Figure S3F and Figure S4E).

### Non-bone marrow CD34 + cells differentiated into fibroblasts to promote fibrosis in cardiac hypertrophy

**Fig. 4:**
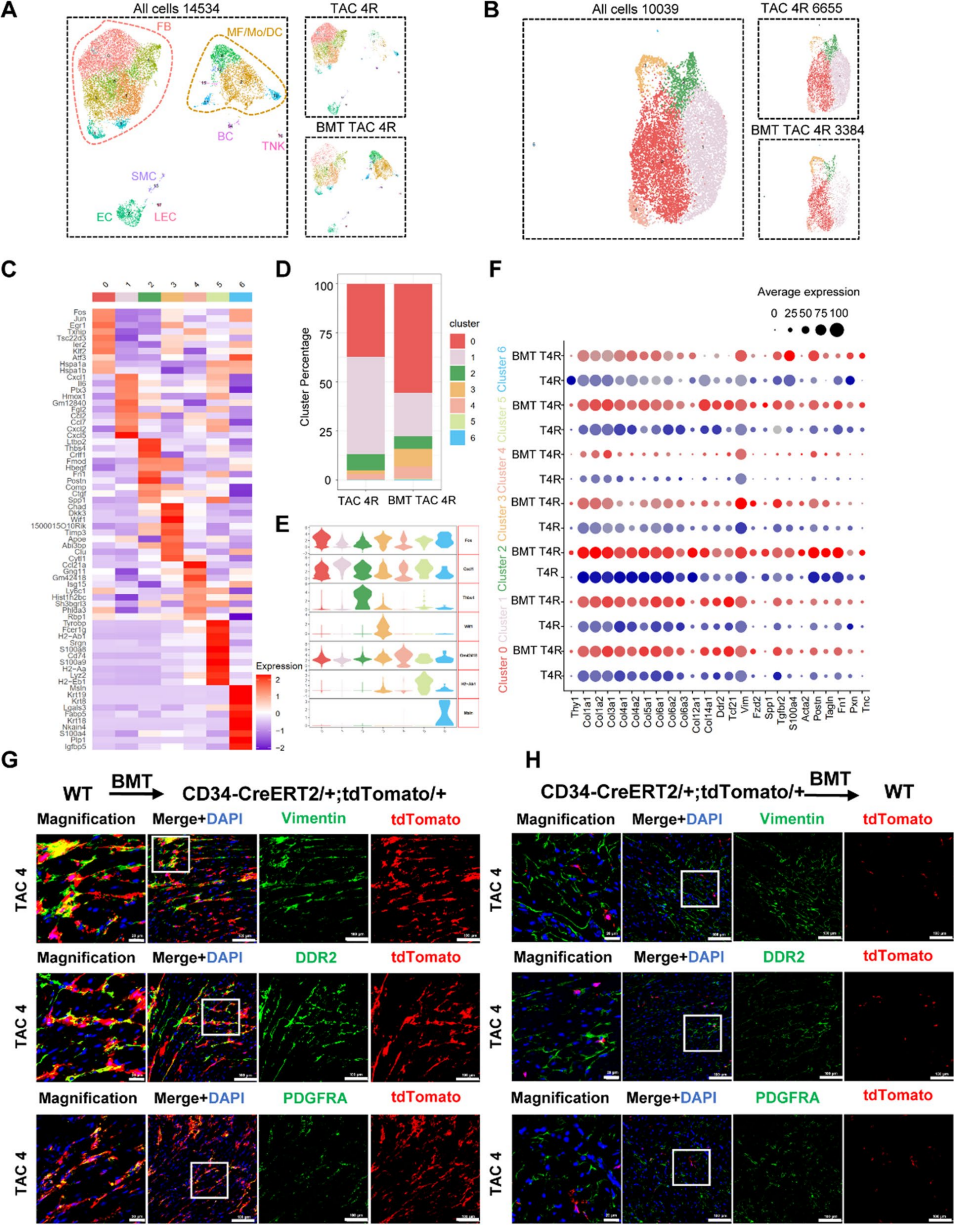
Characterization of non-bone marrow CD34 + cell-derived cells at 4 weeks of pathological cardiac hypertrophy by ScRNA-seq. **A** Umap plot displaying the major cell types and color-coded non-bone-marrow-derived CD34 + cell-derived cells at 4 weeks after TAC surgery. BMT TAC 4R, transplanting bone marrow cells from wild-type C57BL/6 J mice to irradiated CD34-CreERT2;Rosa26-tdTomato, further subjected to TAC surgery; TAC 4 R, normal CD34-CreERT2;Rosa26-tdTomato mice and subjected to TAC surgery. n = 14,534 cells. **B** Umap plot displaying distribution of fibroblast subpopulations in non-bone marrow CD34 + cell-derived cells at 4 weeks after TAC surgery. n = 10,039 cells. resolution = 0.2. **C** Heatmap showing the expression of top ten differentially expressed genes in each cell subclusters. **D** Bar chart showing the percentage of subclusters in datasets between T4 R and BMT T4R group. **E** Violin plot showing the expression of selected marker gene of each subclusters. **F** Dot plot showing the expression of genes related to ECM proteins and fibroblast activation between T4 R and BMT T4R group. **G** Immunofluorescence staining of tdTomato and fibroblast markers (vimentin and DDR2) in bone marrow transplantation of WT (wild-type C57BL/6 J) to CD34-CreERT2;Rosa26-tdTomato mice. n = 7 per group. **H** Immunofluorescence staining of tdTomato and fibroblast markers (vimentin, DDR2 and PDGFRA) in bone marrow transplantation of CD34-CreERT2; Rosa26-tdTomato mice to WT (wild-type C57BL/6 J) (Scale bar = 20 μm and 100 μm). n = 7 per group. BMT, bone marrow transplantation; T4, TAC 4

We next investigated the origin of CD34-derived fibroblast in cardiac hypertrophy. Chimeric mice were created by transplanting bone marrow cells from wild-type C57BL/6 J mice to irradiated *Cd34*-CreERT2;Rosa26-tdTomato, further treated with tamoxifen and subjected to TAC surgery, and harvested 4 weeks after surgery (Additional file 1: Figure S5A). Reconstitution of tdTomato^+^ bone marrow cells was confirmed by flow cytometric analysis (Additional file 1: Figure S5B). To delineate the cell components of non-bone marrow CD34 lineage in cardiac hypertrophy, scRNA-seq was performed on these cells. Total number of 8,290 cells was captured and 6,707 cells passed the quantity control for further analysis. Datasets of TAC 4R (CD34 lineage cells from *Cd34*-CreERT2;Rosa26-tdTomato mice which subjected to TAC surgery for 4 weeks) and BMT TAC 4R (CD34 lineage cells from *Cd34*-CreERT2;Rosa26-tdTomato chimeric mice with wild-type’s bone marrow cells which subjected to TAC surgery for 4 weeks) were aggregated to compare the difference between CD34 lineage and non-bone marrow CD34 lineage cells. Base on the transcriptome features, cells were classified into six major types, including B cells, EC, fibroblast, macrophages, SMC and NK cells (Fig. 4A, B).

Fibroblast was then extracted for further analysis, including 6,655 from TAC 4R group and 3,384 from BMT TAC 4R group, and 7 subclusters were obtained (Fig. 4C, E). Compared with TAC 4R group, multiple subclusters showed remarkable increase in the proportion (Fig. 4D). Cluster 3 (*Wif1*) significantly increased in the BMT TAC 4R group, indicating that non-bone-marrow-derived CD34^+^ cells were likely to be the main source of cluster 3. GO analysis demonstrate that cluster 3 was significantly enriched biological process related to negative regulation of Wnt signaling pathway (Additional file 1: Figure S5C); we speculated that fibroblast-*Wif1* could be a potential target cluster to antagonize cardiac fibrosis development. Cluster similarity analysis revealed that clusters 2 and 3 showed high similarity with clusters identified above (Additional file 1: Figure S5D).

We also found that fibroblast subpopulations in BMT TAC 4R dataset exhibited higher expression of ECM protein-related genes and fibroblast activation in comparison with TAC 4R datasets (Fig. 4F). In addition, immunofluorescence staining was performed to further verify the findings. As shown in Fig. 4G, in the bone marrow transplantation of WT to *Cd34*-CreERT2;Rosa26-tdTomato mice, a large amount of fibroblast markers co-stained with tdTomato, proved that non-bone marrow CD34^+^ cells are the main source to replenish fibroblast pool in cardiac hypertrophy (Fig. 4G; Additional file 1: Figure S5E). On the other hand, in the bone marrow transplantation of *Cd34*-CreERT2; Rosa26-tdTomato to WT mice, the co-staining of fibroblast markers with tdTomato was rarely captured (Fig. 4H; Additional file 1: Figure S5E). In combination with the fluorescence staining data from these two groups in Fig. 4G and H, the results indicated that non-bone marrow CD34^+^ cell-derived fibroblast may be the major participant in cardiac fibrosis. In addition, chimeric mice were created by transplanting bone marrow cells from wild-type C57BL/6 J mice to Cd34-CreERT2;Rosa26-tdTomato. As shown in Figure S5F, the percentage of tdTomato^+^PDGFRA^+^ cell in the heart was almost the same between TAC normal animals (*Cd*34-CreERT2;Rosa26-tdTomato mice without bone marrow transplant) and TAC groups with wild-type BM.

### The role of other non-cardiomyocytes in cardiac hypertrophy

Except from fibroblasts, other non-cardiomyocytes are also crucial participants in cardiac remodeling process. Endothelial cell is another major component of non-cardiomyocytes. We extracted endothelial cells from our datasets and further divided them into nine subclusters (Additional file 1: Figure S6A-C). Gene lists of vascular types identified by Kalucka et al. [33] were used to speculate the identity of the EC subclusters (Additional file 1: Figure S6D). Among all subclusters, cluster 2 was EC-artery, cluster 4 was EC-larger vein and cluster 3 was EC- lymphatic. As shown in Figure S6E, with the development of cardiac hypertrophy, the percentage of cluster 2 showed significantly upregulate, cluster 3 remarkably increased in 4 weeks and decreased in 12 weeks after treatment, indicating the inflammation events were more active in the early stage. Non-bone marrow CD34^+^ cell-derived cluster 3 exhibited remarkably increase compared to total CD34^+^ cell group at the same period, indicating that EC-lymphatic cells were supposed to be non-bone marrow CD34^+^ cell lineage. Flow cytometry demonstrated increasing percentage of ECs marker (CD31) with different stages in total and CD34^+^ cell lineage (Additional file 1: Figure S6F). In addition, Immunofluorescence data also showed that CD34^+^ cell-derived CD31 in 4-week group was significantly higher than that in TAC 0 group (Additional file 1: Figure S6G).

Next, as shown in Figure S6H, in the bone marrow transplantation of wild-type to *Cd34*-CreERT2;Rosa26-tdTomato mice, a large amount of CD31 co-stained with tdTomato. However, in the bone marrow transplantation of *Cd34*-CreERT2; Rosa26-tdTomato to wild-type mice, the co-staining of CD31 with tdTomato was rarely captured, suggesting that non-bone marrow CD34 cells dominantly differentiate into endothelium (Additional file 1: Figure S6H). Cardiac macrophage is a heterogeneous population with high plasticity and adaptability and plays a crucial role in various pathological conditions [43]. Here, we found that macrophages, dendritic cells and monocytes were the major immune cell population in our datasets (Fig. 1D). The population was extracted and further divided into 12 subclusters (Additional file 1: Figure S7A-C). Cluster 1 highly expressed *Lyve1* and *F13a1*, which are recognized as resident macrophage markers [44, 45]. Based on the expression score, cluster 5 was identified as neutrophil; clusters 8 and 11 were identified as dendritic cell (Additional file 1: Figure S7E-F). As shown in Figure S7D, the percentage of resident macrophages decreased in TAC 4 weeks and increased in 12 weeks, indicating that resident macrophages were replaced by circulating derived macrophages. The percentage of cluster 1 in CD34^+^ cell lineage enhanced with the cardiac hypertrophy stages, suggesting that CD34^+^ cell may be an important source of resident macrophage regeneration.

Lymphocytes comprised minor percentage in our datasets; we extracted all lymphocytes of our datasets and divided them into 7 clusters (Additional file 1: Figure S8A and Figure S8C). Based on cell-type-specific marker genes, these clusters were classed into 3 major types, including B cells, T cells and NK cells (Additional file 1: Figure S8B and Figure S8E). NK cells (cluster 2) remarkably increased in TAC 4 groups and decreased in 12 weeks (Additional file 1: Figure S8D).

### Cell–cell communication among cell types during cardiac hypertrophy

To determine the impact of TAC injury on cardiac intercellular signaling, CellPhone DB was used to map ligands and receptors expressed by various cell types (Additional file 1: Figure S9A, Figure S10A). As previously reported [46], fibroblasts were recognized as the most trophic cell population presenting dense connections to multiple cell types. In our datasets, signaling communication by fibroblasts and macrophages composed key features of the interstitial cardiac niche at baseline and TAC groups (Additional file 1: Figure S9A and Figure S10A).

We further analyzed the top ligand–receptor pairs and found several possible intercellular communications between each cell type (Additional file 1: Figure S9B-C, Figure S10 B-C). As shown in Figure S9B, fibroblasts showed elevated expression of several chemokine ligand–receptor pairs with immune cells, indicating that fibroblasts could recruit B cells and macrophage populations in TAC 4-week group (Additional file 1: Figure S9B). In addition, fibroblast highly expressed ECM organization related ligand–receptor pairs in end-stage cardiac hypertrophy, in correspondence to the cardiac remodeling of heart failure (Additional file 1: Figure S9B). Endothelial cell was another cell type exhibiting active cell interactions with other cells (Figure S9C). CXCL12_CXCR4 was highly expressed in each stage of cardiac hypertrophy, indicating that endothelial cells played a crucial role in regulate immune cell chemotaxis even at the baseline stage. PDGFI is reported to be significant for fibroblast migration, PDGFI_LRP1 pair was increased in TAC 4-week group (Additional file 1: Figure S9C). CD74_MIF, CD74_APP and CD74_COPA pairs were significantly enriched by B cells and macrophages at different stages of cardiac hypertrophy (Additional file 1: Figure S10C-D), indicating that CD74 played a crucial role in inflammation activity in cardiac hypertrophy.

### Inducible ablation delineates the role of CD34^+^ cells in cardiac remodeling

Ablation of targeted cells by inducing intracellular expression of diphtheria toxin subunit A (DTA) is a well-established mouse model [47]. To further clarify the role of CD34^+^ cells in myocardial remodeling in response to TAC injury, we then used a CD34 cell depletion system to find out how CD34^+^ cells were involved in myocardial fibrosis. To conditional elimination of CD34^+^ cells in vivo, we generated *Cd34*-CreERT2; Rosa26-eGFP-DTA (Cre/DTA) mice and then pulsed the mice with tamoxifen to induce recombination for DTA expression in CD34^+^ cells.

**Fig. 5:**
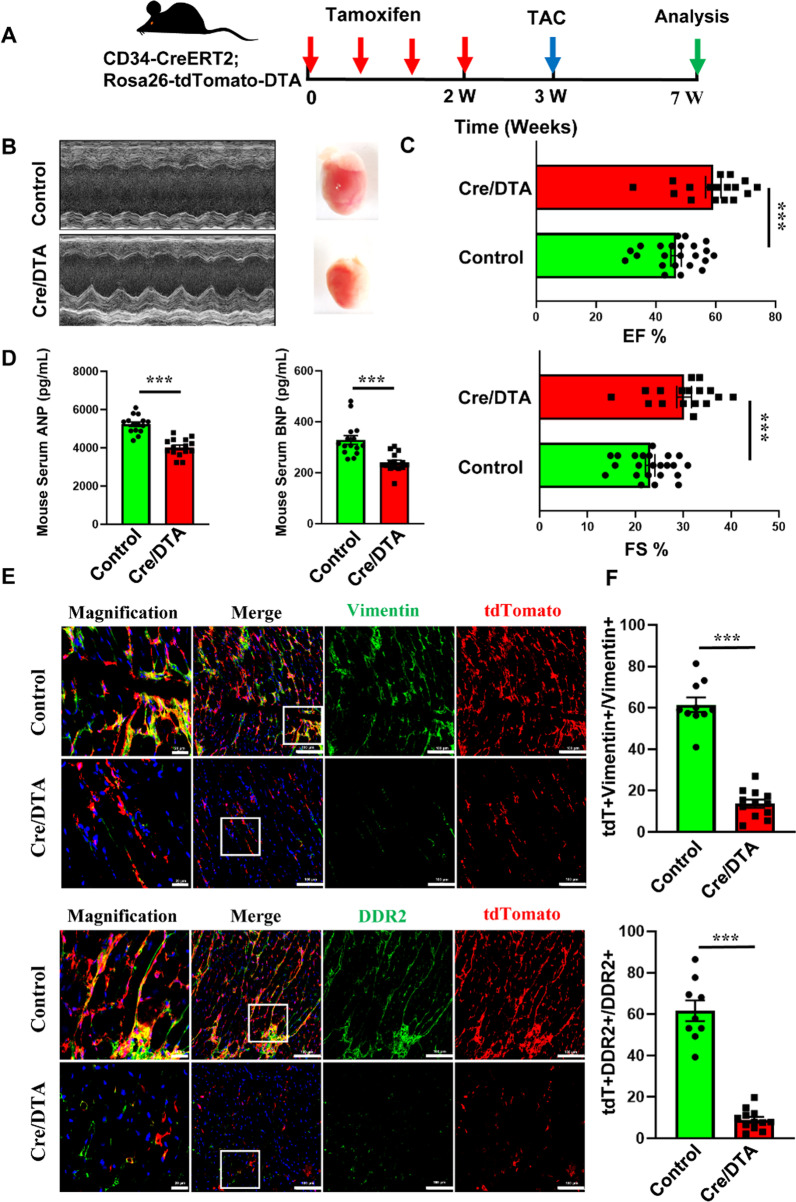
Effect of depletion of CD34 + cells in myocardial fibrosis. **A** Schematic showing CD34-CreERT2;Rosa26-tdTomato/DTA mice. Experimental scheme whereby Cre/DTA mice were given tamoxifen for 1 weeks before TAC 0 or TAC surgery. Heart tissue was harvested 4 weeks after TAC surgery. **B** Representative echocardiography of mice between CD34-CreERT2;Rosa26-tdTomato (Control) and CD34-CreERT2;Rosa26-tdTomato/DTA (Cre/DTA) at 4 weeks after TAC surgery. **C** Echocardiographic measurement of left ventricle ejection fraction (EF) and fractional shortening (FS) of control group and Cre/DTA group after 4 weeks TAC surgery, Data represent mean ± SEM, Cre/DTA group, n = 17; Control group, n = 23. **D** ELISA detection of the serum ANP and BNP between control group and Cre/DTA group after 4 weeks TAC surgery, Data represent mean ± SEM, n = 15 per group. **E** Representative immunostaining images showing staining of tdTomato and vimentin, DDR2 in control group and Cre/DTA group (Scale bar = 20 μm and 100 μm). n = 10 per group. **F** Graph showing percentage of tdTomato expression in vimentin + , DDR2 + fibroblast. Data represent mean ± SEM, n = 10

The surgery was performed on the Cre/DTA mice that were treated with or without tamoxifen, respectively (Fig. 5A); tdTomato^+^ cells were significantly reduced in the Cre/DTA group compared to the control group (Fig. 5E; Additional file 1: Figure S11B). Besides, the left ventricular ejection fraction (LVEF) and fractional shortening were significantly increased, while the levels of ANP and BNP in serum were significantly reduced in the Cre/DTA group, indicating a significant improvement in cardiac function (Fig. 5B–D). Furthermore, H&E, Masson and Sirius red staining on cardiac sections also showed that the severity of fibrosis was also alleviated in the Cre/DTA group (Additional file 1: Figure S11A, Figure S11E). Furthermore, the heart weight was also significantly reduced in the Cre/DTA group compared to the control (Additional file 1: Figure S11D). Immunofluorescence staining data also showed that the expression of *Cd34*-derived fibroblasts (including *Vimentin*, *DDR2* and *PDGFRa*) was significantly reduced in the Cre/DTA group compared to the Control group (Fig. 5E, F; Additional file 1: Figure S11B-C). All these results suggested that CD34^+^ cells were essential for cardiac remodeling and deletion of CD34^+^ cells could alleviate the cardiac fibrotic process and further improve the cardiac function.

### Heart-derived CD34 + cells differentiated into fibroblasts in vitro

**Fig. 6:**
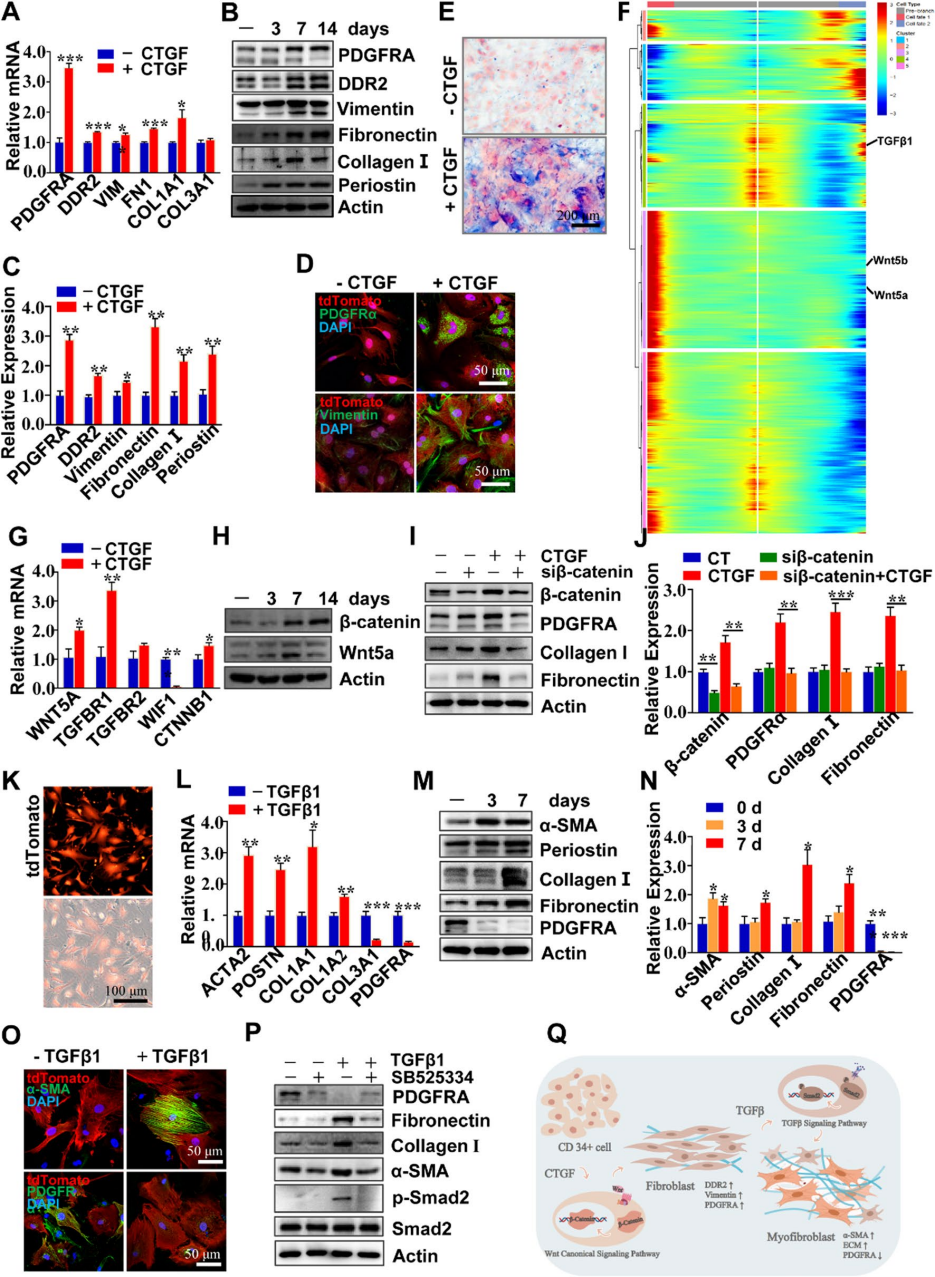
Heart tissue-derived CD34 + cells differentiated into fibroblasts in vitro. **A-E** Heart-derived CD34 + cells treated with 50 ng/mL CTGF for 2 weeks: **A** mRNA levels of fibroblastic markers, GAPDH was used as internal control, n = 6; **B-C** protein expression and quantitative data of fibroblastic markers and ECM proteins after treated with CTGF for 2 weeks, n = 5. **D** immunofluorescence staining of PDGFRα and vimentin in CD34 + cell (Scale bar = 50 μm). **E** Masson's trichrome staining shows collagen deposition in CD34 + cells with CTGF treatment(Scale bar = 200 μm). **F** Heatmap of the significantly changed genes (*P* < 0.01) discovered by the BEAM function from monocle in the branch point 3 in Fig. 3F. Cd34 and Ly6a genes were detected in Gene Module 1, wnt pathway-related genes were detected in module 3 and TGFβ pathway related genes in module 4. **G** Genes expression of Wnt (WNT5A, WIF1, CTNNB1) and TGFβ receptor (TGFBR1,TGFBR2) pathway in CD34 + cells with or without CTGF (50 ng/mL) stimulation at the 2-week time point, n = 6. **H** Protein (β-catenin, Wnt5a) expression in CD34 + cells with or without CTGF (50 ng/mL) treatment for 3, 7, and 14 days, n = 5. **I-J** CD34 + cells were transfected with β-catenin siRNA (10 nM) and treated with CTGF (50 ng/mL) for 2 weeks, fibroblastic markers were determined by western blotting and quantitative data was shown, n = 4. **K** TdTomato positive cells were isolated from the heart of TAC 12-week mice (Scale bar = 100 μm). **L-N** TdTomato positive cells were stimulated with TGFβ1(5 ng/mL), mRNA levels of ACTA2, POSTN, COL1A1, COL1A2, COL3A1 and PDGFRA were presented upon 7 days of TGFβ1 treatment. proteins were determined by western blotting and quantitative data was shown, n = 3. **O** Immunofluorescence staining of α-SMA and PDGFRα in tdTomato positive cells upon 7 days of TGFβ1 stimulation (Scale bar = 50 μm). **P** Myofibroblastic marker (α-SMA), fibroblastic markers (periostin, PDGFRA), and ECM proteins (collagenI, fibronectin) expression in tdTomato positive cells, which TGFβ1 receptor activity was inhibited by SB525334 (2 μM), at the 7-day time point of TGFβ1 treatment. **Q** Schematic of proposed origin of fibroblast and myofibroblast in heart failure, *P < 0.05; **P < 0.01; ***P < 0.001

Our data so far suggest a possible role of resident CD34^+^ cells in fibroblast generation. Primary tdTomato + cells isolated from the heart of TAC-induced heart failure mice exhibited high heterogeneous both in cell size and cell morphology in comparison with CD34^+^ cells isolated from the TAC 0 control mice (Additional file 1: Figure S12B). We supposed that vascular CD34^+^ cells may have higher potential to differentiate into fibroblastic cells, To address the question, heart residential CD34^+^ cells were isolated from the *Cd34*-CreERT2;Rosa26-tdTomato mice (see Methods) and verified with CD34 immunofluorescence staining (Additional file 1: Figure S12E). Then CD34^+^ cells were treated with CTGF, a growth factor sufficient to differentiate MSCs into fibroblast cells [32]. We found that a broad array of fibroblastic hallmarks m such as *PDGFRα*, *DDR2*, *Vimentin*, *Periostin* (*Postn*) and some ECM proteins fibronectin, collagen I were up-regulated in CD34 + cells treated with CTGF over 2 weeks (Fig. 6A–D). Masson staining also showed the collagen deposition in CD34^+^ cells upon CTGF stimulation (Fig. 6E). These results indicate that heart residential CD34^+^ cells can be directed differentiation to fibroblastic cells by CTGF in vitro. To further elucidate the underlying mechanism, we analyze the scRNA-seq data which revealed that Wnt and TGFβ pathways may have a pivotal role during this process (Fig. 6F, Additional file 1: Figure S12G). CD34^+^ cells subjected to CTGF stimulation expressed higher levels of Wnt5a and β-catenin, of which proteins promote Wnt pathway, while we decreased the expression of WIF1 which inhibits Wnt signaling (Fig. 6G, H). TGFBR11 was also up-regulated in CD34^+^ cells with CTGF treatment (Fig. 6G). It has been shown that β-catenin plays a crucial role in heart failure [48]; thus, we wondered the way of β-catenin during the differentiation procession. siRNA for β-catenin was transfected into CD34 + cells to down-regulate β-catenin expression, strikingly, both fibroblastic cell markers (*PDGFRα*, *Periostin*) and ECM proteins (fibronectin, collagen I) were significantly decreased in CD34^+^ cells induced by CTGF stimulation (Fig. 6I-J; Additional file 1: Figure S12F), suggesting that Wnt-β-catenin pathway is involved in the process of CD34^+^ cells differentiation into fibroblastic cells (Fig. 6Q).

As we know that the fibroblasts would undergo differentiation into the myofibroblasts once they are stimulated mechanically or chemically, and smooth muscle actin (α-SMA) is regarded as a main hallmark of myofibroblasts [49]. TdTomato^+^ cells that were isolated from heart failure mouse (Fig. 6K) expressed higher levels of fibroblastic markers and ECM proteins but less levels of CD34 (Additional file 1: Figure S12A, S12C and S12D). Thereafter we treated these “fibroblast-like” tdTomato^+^ cells with TGFβ1 to induce differentiation into myofibroblasts. Remarkably, TGFβ1-treated tdTomato^+^ cells exhibited higher α-SMA synthesis, compared with those tdTomato^+^ cells without TGFβ1 stimulation (Fig. 6L–O). Meanwhile, ECM proteins were still present (Fig. 6L–N), whereas PDGFRα was dramatically down-regulated (Fig. 6L–O) in tdTomato^+^ cells upon TGFβ1 stimulation. Furthermore, SB525334, a selective TGFβ1 receptor inhibitor, was used to block TGFβ1 signaling. We found that TGFβ1 induced the expression of α-SMA and ECM proteins were profoundly alleviated by SB525334 (Fig. 6P), consistently Smad2 activation was also inhibited in this process. This in vitro finding was confirmed by in vivo ELISA test showing elevated TGFβ1 concentration in TAC heart that was inhibited by SB525334 (Figure S12H), which suggests that differentiation from CD34-derived tdTomato^+^ cells into myofibroblasts is dependent on TGFβ1/Smad2 pathway (Fig. 6Q).

### Cell atlas of heart tissues in patients

**Fig. 7:**
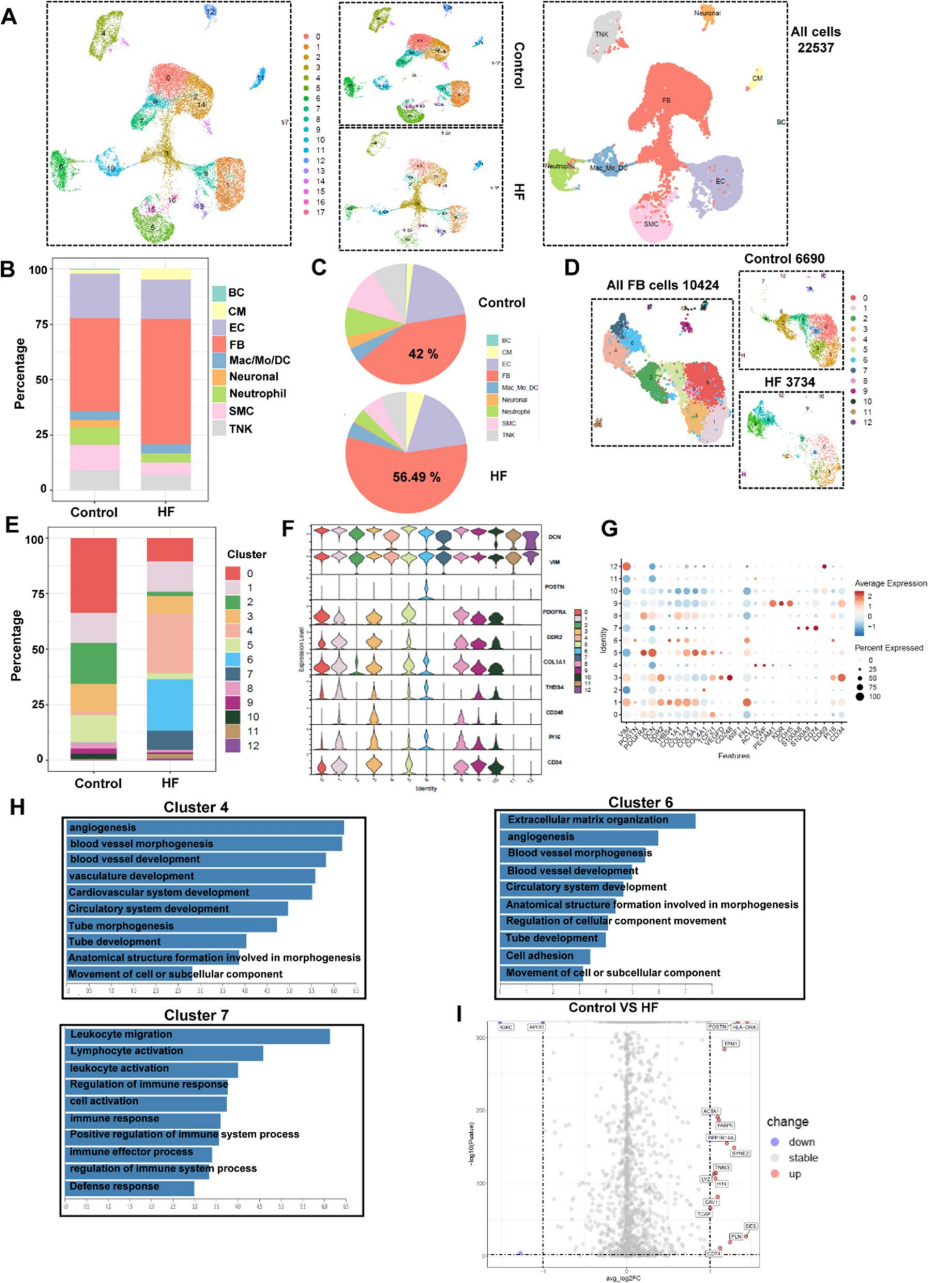
Cell composition of human heart.** A** UMAP plot displaying the major cell types and color-coded cell clusters of human heart, n = 22,537 cells. (Control: normal heart; HF: Heart Failure). **B** Bar chart showing the percentage of major cell types among human heart (Control: normal heart; HF: Heart Failure). **C** Pie plot showing the percentage of major cell types among human heart between control and heart failure. **D** UMAP plot displaying distribution of fibroblast subpopulations between control and heart failure group, n = 10,242 cells, resolution = 0.5. **E** Bar chart showing the percentage of subclusters in datasets between control and diseased heart (HF). **F** Violin plot showing the expression of selected representative cell marker genes in each cell cluster. **G** Dot plot showing the expression of selected fibroblast cell marker genes in each cell cluster. Dot size reflects the percentage of cells expressing the selected gene in each cell cluster. **H** Bar plot showing the GO datasets score among fibroblast subclusters. **I** Volcano plot showing the DEGs between control and heart failure (p-value < 0.01 and log2FC > 1 was labeled)

Next, we intended to determine whether similar changes occur on patients with heart failure. To determine cellular landscape of human cardiac non-cardiomyocytes in heart failure, we obtained single-cell transcriptomes for 22,537 cells by integrating two groups from human heart (Control: normal heart; HF: Heart failure) by strict quality control (Additional file 1: Figure S13A), and defined 9 major cell type which included fibroblasts, endothelial cells, cardiomyocytes, TNK cells, macrophages, B cells, neuronal cells, neutrophils and smooth muscle cells, based on their respective molecular signatures (Fig. 7A; Additional file 1: Figure S13B-C). Furthermore, the results of scRNA-seq data of human hearts were consistent with those of mice; we observed the proportion of fibroblasts was significantly higher in heart failure than the control group (Fig. 7B, C). Several subpopulations of fibroblasts such as fibroblast-THBS4, fibroblast-FN1, fibroblast-POSTN were also found in human heart increasing with heart failure (Fig. 7 G; Additional file 1: Figure S13D), which is consistent with our results in mice.

Similarly, these fibroblasts were partitioned into distinct subtypes comprising cells from all the groups. The dispersion of fibroblast clusters in the UMAP plots indicated a high degree of fibroblast heterogeneity (Fig. 7D). Notably, compared with the control groups, several distinct fibroblast subtypes show significant changes in constitution percentage in the heart failure groups (Fig. 7E). Activation of fibroblasts was evidenced by increased proportions of cluster 4, cluster 6 and cluster 7 at the stage of heart failure (Fig. 7E). GO analysis demonstrated that clusters 4 showed the elevated capacity of angiogenesis and blood vessel development involved in fibrosis development (Fig. 7F–H). Since cluster 4 enriched the expression of genes (VWF, PECAM1) related to EC function, we defined it as a subcluster undergoing endothelial to mesenchymal transition (EndoMT). Consistent with our mouse data, cluster 6 (subtypes with high expression of extracellular matrix protein including POSTN, THBS4) at heart failure which showed elevated capacity of ECM organization, extracellular structure organization (Fig. 7F–H). In addition, GO analysis demonstrated that cluster 7 showed elevated capacity of lymphocyte activation and regulation of immune response, and it also with the high expression of genes (S100A8, CD74, CD69). Thus, we defined it as a fibroblast subcluster related to immune response (Fig. 7F–H). Among all the subclusters, further GO enrichment analysis showed that extracellular structure organization and vasculature development were highly enriched in CD34^hig h^ fibroblasts compared with the CD34^low^ fibroblasts (cluster 3); in addition, this cluster also showed elevated expression of PI16 (progenitor cell markers) and CD248 (Fig. 7F, G; Figure S13E), also indicating that CD34^high^ fibroblasts may exhibit specific functions during cardiac homeostasis and after injuries, which were consistent with our results from single-cell RNA sequencing data in mice.

**Fig. 8:**
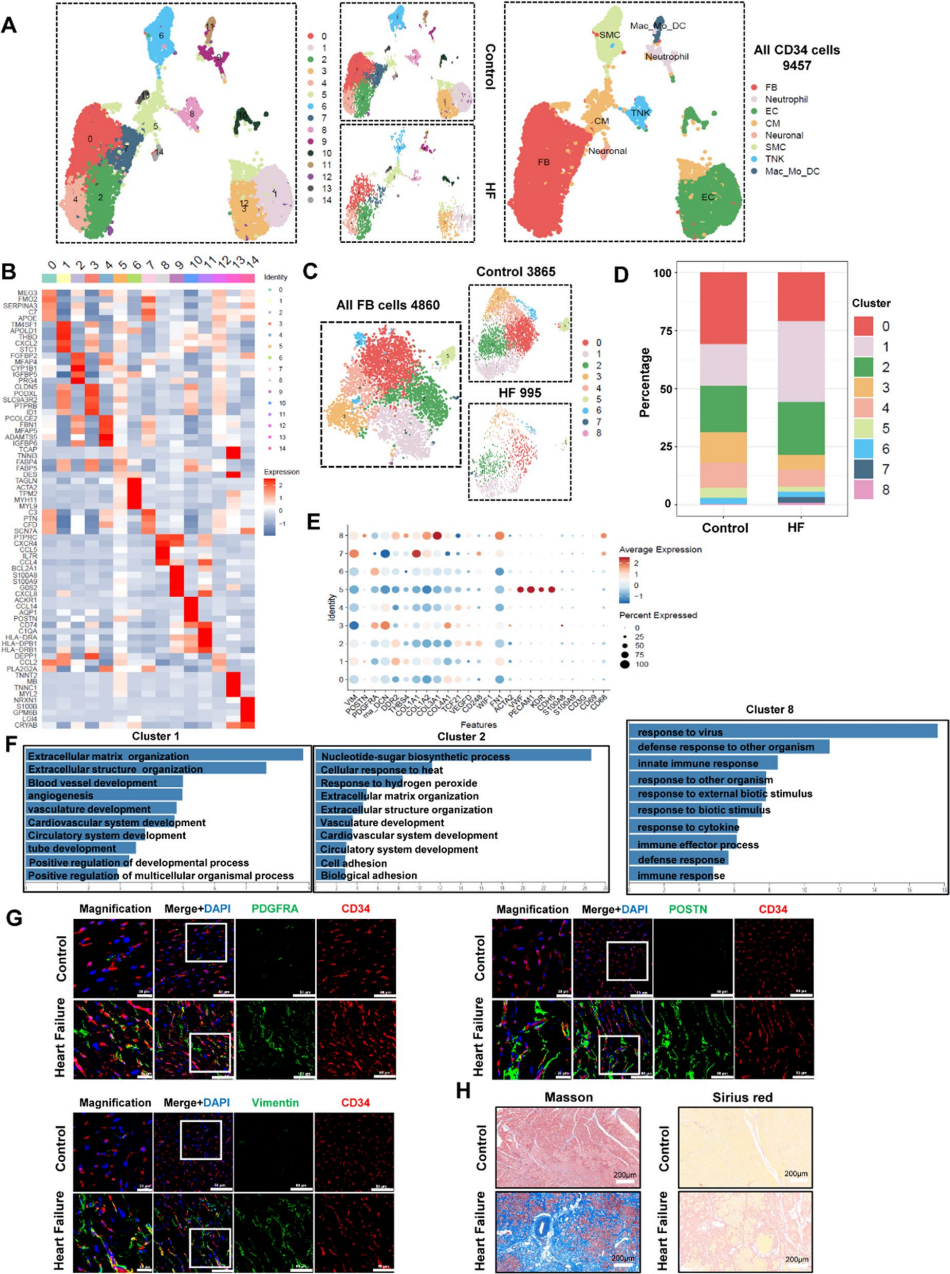
Characterization of CD34 + cells in human hearts**. A** UMAP plot displaying the major cell types and color-coded CD34 + cells of human heart with the disease, n = 9,457 cells. **B** Heatmap showing the expression of top five differentially expressed genes in each cell subcluster. **C** UMAP plot displaying distribution of fibroblast subpopulations between control and diseased heart, n = 4,860 cells, resolution = 0.5. **D** Bar chart showing the percentage of subclusters in datasets between control and diseased heart. **E** Dot plot showing expression levels of top five differentially expressed genes in each cell cluster. Dot size reflects the percentage of cells expressing the selected gene in each cell cluster. **F** Bar plot showing the GO datasets score among fibroblast subclusters. **G**. Representative immunostaining images showing staining of CD34 and vimentin, PDGFRA and POSTN in human heart (scale bar = 20 μm and 100 μm). **H** Sirius red and Masson staining showing different degrees of fibrosis between normal heart (control) and heart with heart failure (HF)

Up-regulated differentially expressed genes (DEGs) of different datasets showed that the heart failure groups expressed significantly genes involved in cardiac fibrosis, including POSTN and DES (Fig. 7I). Consistently with the mouse heart data, immunofluorescence staining data showed that the expression of VIM, PDGFRA and POSTN were significantly increased in the heart failure group compared with the normal heart (Fig. 8G). In addition, Masson and Sirius red staining also showed that the area of fibrosis in the heart with heart failure is much more severe than that in the normal heart (Fig. 8H).

### CD34^+^ cells in patients with heart failure

In order to delineate the constitution map of human cardiac resident CD34 lineage cells during heart failure, we extracted the cells that expressing CD34 from the human single-cell RNA sequencing data, which were CD34^+^ cells. We then performed scRNA-seq in CD34 lineage between control- and heart failure-groups, total number of 9,457 cells were captured and passed the quantity control for further analysis. Based on the transcriptome characteristics, cells are divided into several major cell types, e.g., fibroblasts, endothelial cells and macrophages (Fig. 8A, B; Additional file 1: Figure S14A-B). Based on the transcriptome characteristics, fibroblast was then extracted for further analysis to discover their heterogeneous features, including 4,860 cells from both group and 9 subclusters were obtained. Furthermore, we observed the proportion of markers associated with fibroblasts (VIM, POSTN, DDR2, THBS4, COL1A1, COL1A2, FN1) were significantly higher in heart failure than the control group in the human data (Additional file 1: Figure S14C), which were consistent with that in mice.

In order to illustrate the CD34 lineage fibroblast heterogeneous features, the fibroblasts were then extracted from CD34 lineage cells for further analysis, including 4,860 cells from both groups and 9 subclusters were obtained (Fig. 8C, D). Compared with control group, several subclusters (clusters 1, 2, 7 and 8) showed remarkable increase in the proportion in heart failure group (Fig. 8D). Consistent with above data, cluster 1 (subtypes with high expression of extracellular matrix protein including DDR2, THBS4, FN1, POSTN) at heart failure which involved in ECM organization, extracellular structure organization (Fig. 8E, F). In addition, cluster 8 (with the high expression of COL3A1, FN1 and CD68) was associated with inflammatory response by GO analysis. Thus, we defined it as a fibroblast subcluster related to immune response (Fig. 8E, F). In addition, just as we found a new population of fibroblasts (VEGFD) in mouse CD34 lineage fibroblasts, we also found this new subpopulation (cluster 2 with high expression of VEGFD and CD248) in human CD34 lineage fibroblasts, and GO analysis demonstrated that cluster 2 is involved in extracellular matrix formation and vascular development (Fig. 8E, F). This further confirms what we found in mice. Similarly, CD34 co-stained with vimentin, PDGFRA and POSTN were also increased (Fig. 8G). Taken together, patients with heart failure displayed a marked alterations of cell landscape in heart tissues implicating a contribution of CD34^+^ cells to fibrosis. Therefore, we further identified CD34^+^ cells in humans, at least a subpopulation of CD34^+^ cells in the heart, has a progenitor cell-like phenotype, which could transdifferentiate into multiple cell types especially the fibroblasts.

## Discussion

In the present study, we uncovered the heterogeneity of non-cardiomyocytes, especially CD34^+^ cells in the heart, and provided the evidence that CD34^+^ cells can differentiate into various types of cells, including immune and endothelial cells and fibroblasts by using scRNA-seq and lineage tracing techniques. All of them could contribute to the process of myocardial remodeling. Furthermore, depletion of CD34^+^ cells in the mouse model of heart failure leads to decreased myocardial fibrosis and improved cardiac function. Interestingly, we found that CD34^+^ cells isolated from the heart differentiate to fibroblasts via the TGFβ1/Smad2 pathways. Importantly, we provided cell atlas of human hearts with the disease that shares several similarities to the findings of animal models, e.g., subpopulations of fibroblasts expressing CD34 cell marker. These findings provided the first evidence that CD34^+^ cells not only regenerate endothelial cells for angiogenesis, but also differentiate into an active population of fibroblasts enhancing cardiac fibrosis.

Recent studies have shown that non-cardiomyocyte cells (including endothelial cells, inflammatory cell, fibroblasts) play an important role in myocardial remodeling [50–52]. Several findings suggest that changes in endothelial cell-related genes have a significant impact on myocardial remodeling [53–56]. Although fibroblasts predominantly participated in ECM organization during myocardial remodeling, endothelial dysfunction is substantially correlated to ECM remodeling in heart failure. Our data indicate that CD34^+^ cells can directly differentiate into endothelial cells during heart remodeling, which showed an altered gene expression, especially pro-inflammatory cytokines. It was found that CXCL12_CXCR4 was highly expressed in each stage of cardiac hypertrophy, indicating that endothelial cells played a crucial role in myocardial remodeling by secreting some chemokines and cytokines and regulating immune cell chemotaxis in heart failure. In addition, endothelial cells also secret multiple pro-inflammatory cytokines, which are crucial for immune cell adhesion and infiltration enhancing pathological hypertrophy [50]. The expression of PDGF_LRP1 pair, which is reported to be significant for fibroblast migration, was significantly increased in 4-week group. These observations lead us to propose that CD34^+^ cell-derived endothelial cells might be less mature appearing as dysfunctional endothelium expressing pro-inflammatory genes regulating heart remodeling via enhanced inflammatory response.

Growing number of studies have shown that inflammatory response is an important factor in the development of heart failure [26, 57]. Cardiac macrophage is a heterogeneous population with high plasticity and adaptability; macrophages also play a critical role in regulation of fibrotic responses in many different tissues [43, 50]. Resident macrophages are known to respond to micro-environmental cues by modulating synthesis of cytokines and growth factors [58] and produce large amounts of pro-fibrotic growth factors [50], which can regulate fibrosis. In our study, by detailed analysis of tdTomato^+^ macrophage population, the percentage of resident macrophage in CD34^+^ cell lineage significantly increased in the progression of hypertrophy and heart failure. This result indicates that CD34^+^ cells are a main source of cardiac-activated resident macrophages that may contribute directly to the fibrotic process.

In our scRNA-seq data, we noticed that cluster 13 expressing cardiomyocyte marker genes (e.g., Ankrd1, Mb, Tnnt2), although we tried to isolate non-cardiomyocyte cells for the experiments. A possible explanation is that a small proportion of cardiomyocytes were included as well during the cell isolation. Similarly, our protocol used to isolate non-cardiomyocytes cannot obtain all the cells in a proper ratio as presented in vivo. We have to point out thus limitation of cell isolation for single-cell RNA-sequencing analysis.

Cardiac fibrosis is one of the most important causes of myocardial remodeling [59, 60]. The severity of fibrosis is correlated with the progression of heart failure [61]. However, due to the lack of cell-specific marker, previous study and analysis of fibroblasts are limited by imprecise definitions for this cell type. On the basis of mouse scRNA-seq data, several subpopulations of fibroblasts, e.g., fibroblast-*Thbs4*, fibroblast-*Wif1*, fibroblast-*Cd248* and fibroblast-vegfd, were found in hypertrophy heart. By combination of single-cell RNA-seq and linear tracing analyses, we found that CD34^+^ cells can differentiate into fibroblasts composed of several subpopulations mentioned above during myocardial remodeling. Fibroblast-*Cd248* showed elevated expression of multiple stem cell markers, and GO analysis exhibited several functions related to stem cell/progenitor cells. To our surprise, the results of scRNA-seq data of human hearts were consistent with those of mice, several subpopulations of fibroblasts such as fibroblast-THBS4, fibroblast-CD248, fibroblast-PDGFRA, fibroblast-DDR2 and fibroblast-POSTN were also found in human heart with heart failure. Besides, we further analyzed the CD34^+^ cells from the human heart and found that CD34^+^ cells can differentiate into fibroblasts composed of several subpopulations mentioned above during myocardial remodeling. And GO analysis exhibited several functions related to ECM organization, extracellular structure organization, angiogenesis, blood vessel morphogenesis, blood vessel development and immune response which are involved in fibrosis development. Therefore, these data suggest that CD34^+^ cells may participate in fibrosis by differentiating into active fibroblasts expressing a set of surface markers that distinguish from other cardiac fibroblasts during myocardial remodeling, indicating the heterogeneity and the functional diversity of cardiac fibroblasts for fibrosis.

As described above, a novel observation in mouse models is that CD34^+^ cells can differentiate into active fibroblasts that participate in cardiac fibrosis. It is essential to confirm them in vitro results and clarify the mechanisms of cell differentiation and activation. Our in vitro data displayed that CD34^+^ cells from normal `heart can differentiate into fibroblasts expressing a panel of fibroblastic markers with collagen production in response to CTGF stimulation. Furthermore, pseudotime analysis of CD34^+^ cells and in vitro study displayed a critical role of TGFβ/Wnt-β-catenin pathway in regulating cell differentiation into fibroblasts. In addition, in our vitro study, we also isolated tdTomato^+^ cells from the hearts of treated mice. In response to TGFβ1, tdTomato^+^ cells further differentiate into myofibroblasts via TGFβ–Smad2 signaling pathways. These results suggest that naïve CD34^+^ cells can differentiate into fibroblasts that can become myofibroblasts in response to TGFβ1 stimulation.

Recently, it was reported that genetic ablation of cardiac fibroblasts after hypertensive or ischemic injury has been shown to reduce fibrosis and improve heart function in mice [62–64]. Clinically, no therapeutic technique is available to directly target excessive fibrosis, and very few interventions have been shown to improve cardiac function and clinical outcomes in patients with impaired cardiac compliance in heart failure. To clarify the role of CD34^+^ cells in myocardial remodeling in response to pressure overload, the surgery was performed on the Cre/DTA animal models. Surprisingly, our data show that partially depletion of CD34^+^ cells resulted in a reduction of fibrotic area and a significant improvement in cardiac function compared with the control animals. When CD34^+^ cells were partially eliminated, the fibroblasts in the heart were markedly reduced, leading to a decrease in myocardial fibrosis and improving cardiac function. Taken together, results support the notion that it could be possible to target CD34^+^ cells directly in the treatment of myocardial fibrosis.

Treatment of heart failure with CD34^+^ cells from bone marrow has been carried out for decades [65]. CD34^+^ cells were collected from bone marrow and then injected into the heart. The outcome of clinic trials is variable and controversial [12, 18, 22–24]. In the present study, we used single-cell RNA-seq and lineage tracing techniques providing the direct evidence that bone marrow CD34^+^ cells cannot differentiate into endothelial cells but rather inflammatory cells. Human sample immunostaining indicates that CD34^+^ cells in the heart tissue present in both immune cells and fibroblasts. These results implicate that bone marrow CD34^+^ cells can only become immune/inflammatory cells in vivo and non-bone marrow CD34^+^ cells have an ability to differentiate into endothelial and fibroblasts. Thus, we have to reconsider an alternative strategy using CD34^+^ cell therapy for patients.

## Conclusions

In summary, with scRNA-seq of hearts from both mouse and human and lineage tracing, we demonstrated a critical role of CD34^+^ cells in the process of myocardial remodeling, managed to systematically characterize the cellular landscape of myocardial fibrosis at a single-cell resolution and provide a comprehensive cell atlas including CD34^+^ cells, fibroblasts, endothelial cells and immune cells. We further validated that bone marrow-derived CD34^+^ cells cannot differentiate into endothelial cells in pressure overload model. Partial depletion of CD34^+^ cells resulted in a reduction in fibrosis and a significant improvement in cardiac function. Besides, Wnt-β-catenin and TGFβ1/Smad pathways are crucial in the differentiation of CD34^+^ cells into myofibroblasts. Overall, our study not only provides a wealth of reference information on cell types and interaction networks during the heart failure, but also offers novel insights into the pathogenesis of cardiac fibrosis and possibilities for the development of therapeutic approach for heart failure in the future.

## Supplementary Information

**Additional file 1. Figure S1.** Quality control of single-cell RNA sequencing and comparison of single-cell RNA sequencing data of different normal heart. **Figure S2.** GO and trajectory analysis of selected subclusters of fibroblasts. **Figure S3.** Construction and verification of CD34-CreERT2; Rosa26-tdTomato lineage tracing mouse. **Figure S4.** Top five genes expressed and GO analysis of subclusters of fibroblasts in CD34 lineage cells. **Figure S5.** Schematic depicting of bone marrow transplantation and confirmation of the reconstitution of bone marrow cells. **Figure S6.** Characterization of EC clusters of different stages of pathological cardiac hypertrophy by ScRNA-seq. **Figure S7.** Characterization of MF/Mo/DC clusters of different stages of pathological cardiac hypertrophy by ScRNA-seq. **Figure S8.** Characterization of Lymphocytes clusters of different stages of pathological cardiac hypertrophy by ScRNA-seq. **Figure S9.** Cell communication between different cell types at different stages of pathological cardiac hypertrophy. **Figure S10.** Cell communication between different cell types at different stages of pathological cardiac hypertrophy. **Figure S11.** Effect of depletion of CD34+ cells on myocardial fibrosis. **Figure S12.** Heart-derived CD34+ cells can be directed into fibroblasticcells. **Figure S13.** Quality control of human heart single-cell RNA sequencing and the respective molecular signatures of each subcluster. **Figure S14.** Top five genes of each cluster and the expression of selected cell marker gene in CD34+ cells ofhuman heart.

## Data Availability

All data needed to evaluate the conclusions in the paper are present in the paper and/or the Supplementary Materials. The scRNA-seq data of our study are available for reproducing the results, which are being uploaded. The data that support the findings of this study are openly available in Gene Expression Omnibus (GEO) (GSE198833) (web links: https://www.ncbi.nlm.nih.gov/geo/query/acc.cgi?acc=GSE198833); The single-cell RNA-sequencing publicly available data of human used in this study are available in the GSA-Human database under the accession code HRA002066 (web links: https://bigd.big.ac.cn/gsa-human/browse/HRA002066) and the Control heart data in HRA001765 ( web links: https://bigd.big.ac.cn/gsa-human/browse/HRA001765) and also in the Gene Expression Omnibus (GEO) (GSE222144) (https://www.ncbi.nlm.nih.gov/geo/query/acc.cgi?acc=GSE222144). The human dataset in GSA-Human is available under restricted access, while the dataset in GEO is not.

## Abbreviations

scRNA-seq: Single-cell RNA sequencing
TAC: Transverse aortic constriction
DTA: Diphtheria toxin fragment A
BMT: Bone marrow transplantation
LVEF: Left ventricular ejection fraction
FS: Fractional shortening
RT-qPCR: Real-time quantitative polymerase chain reaction
postn: Periostin
α-SMA: Smooth muscle actin
Control: Normal heart
HF: Heart failure
EndoMT: Endothelial-to-mesenchymal transition
DEGs: Differentially expressed genes
