# Supplementary material for: Single cell and lineage tracing studies reveal the impact of CD34+ cells on myocardial fibrosis during heart failure
Source: Stem Cell Res Ther. 2023 Feb 20;14:33. doi: 10.1186/s13287-023-03256-0 (PMC9942332; doi:10.1186/s13287-023-03256-0)
Supplement: Supplementary file 1 — Additional file 1. Figure S1. Quality control of single-cell RNA sequencing and comparison of single-cell RNA sequencing data of different normal heart. Figure S2. GO and trajectory analysis of selected subclusters of fibroblasts. Figure S3. Construction and verification of CD34-CreERT2; Rosa26-tdTomato lineage tracing mouse. Figure S4. Top five genes expressed and GO analysis of subclusters of fibroblasts in CD34 lineage cells. Figure S5. Schematic depicting of bone marrow transplantation and confirmation of the reconstitution of bone marrow cells. Figure S6. Characterization of EC clusters of different stages of pathological cardiac hypertrophy by ScRNA-seq. Figure S7. Characterization of MF/Mo/DC clusters of different stages of pathological cardiac hypertrophy by ScRNA-seq. Figure S8. Characterization of Lymphocytes clusters of different stages of pathological cardiac hypertrophy by ScRNA-seq. Figure S9. Cell communication between different cell types at different stages of pathological cardiac hypertrophy. Figure S10. Cell communication between different cell types at different stages of pathological cardiac hypertrophy. Figure S11. Effect of depletion of CD34+ cells on myocardial fibrosis. Figure S12. Heart-derived CD34+ cells can be directed into fibroblasticcells. Figure S13. Quality control of human heart single-cell RNA sequencing and the respective molecular signatures of each subcluster. Figure S14. Top five genes of each cluster and the expression of selected cell marker gene in CD34+ cells ofhuman heart. [file 13287_2023_3256_MOESM1_ESM.pdf]

Supplementary Materials

Figure S1. Related to Figure 1. Quality control of single-cell RNA sequencing and comparison of single-cell RNA sequencing data of different normal heart.

Figure S2. Related to Figure 2

Figure S3. Related to Figure 3

Figure S4. Related to Figure 3

Figure S5. Related to Figure 4

Figure S6. Characterization of EC clusters of different stages of pathological cardiac hypertrophy by ScRNA-seq.

Figure S7. Characterization of MF/Mo/DC clusters of different stages of pathological cardiac hypertrophy by ScRNA-seq.

Figure S8. Characterization of Lymphocytes clusters of different stages of pathological cardiac hypertrophy by ScRNA-seq.

Figure S9. cell communication between different cell types at different stages of pathological cardiac hypertrophy.

Figure S10. cell communication between different cell types at different stages of pathological cardiac hypertrophy.

Figure S11. Effect of depletion of CD34+ cells on myocardial fibrosis

Figure S12. Heart-derived CD34+ cells can be directed into fibroblastic cells.

Figure S13. Related to Figure 7.

Figure S14. Related to Figure 8.

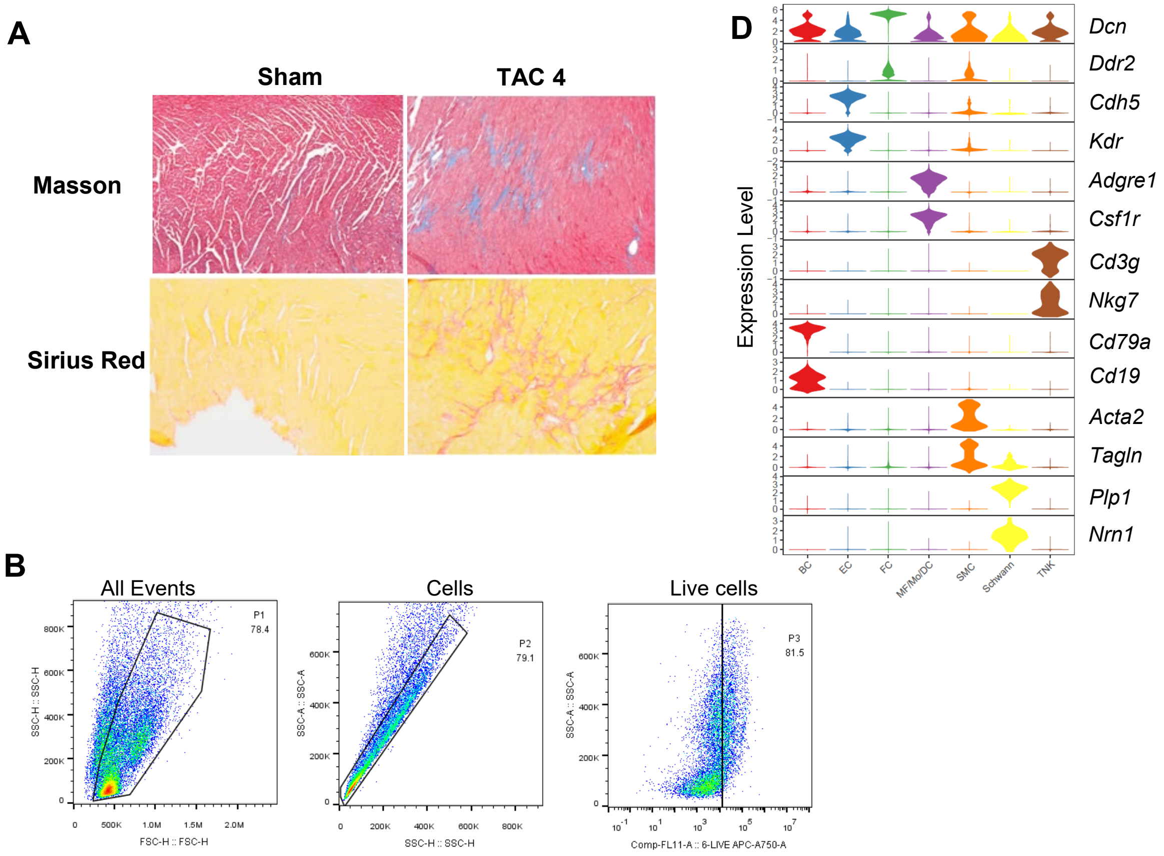

C

	TAC 0	TAC 4	TAC 12	TAC0R	TAC 4R	TAC 12R	BMT TAC 4R
Estimated Number of Cells	7057	8950	10124	10,165	10197	15171	8290
Mean Reads per Cell before Aggregation	95,853	102,475	115,224	117,789	101,218	112,263	92,987
Median Genes per Cell before Aggregation	2,721	2,361	3253	3,489	2360	3,405	2,972
Fraction of Reads Kept in Aggregation	100.0%	88.6%	87.8%	78.5%	92.1%	86.1%	95.9%
Pre-Normalization Total Reads per Cell after Aggregation	95,853	102,475	115,224	117,789	101,218	112,263	92,987
Pre-Normalization Confidently Mapped Barcoded Reads per Cell after Aggregation	59,364	66,999	67,644	75,668	64,439	68,913	61,909

Figure S1. Related to Figure 1. Quality control of single-cell RNA sequencing and comparison of single-cell RNA sequencing data of different normal heart. (A). Masson staining (Blue) and Picro-Sirius red staining (Red) of heart sections to evaluate the level of fibrosis. **(B)**. Representative gating strategy for sorting live nucleated (Hoechst+Dead Cell Stain) single cells for scRNA-seq. **(C)**. Basic quality control metrics of indicated datasets before and after cell ranger aggregation and filtering. **(D)**. Violin plot showing the expression of selected cell marker gene to define cell clusters from integrated data.

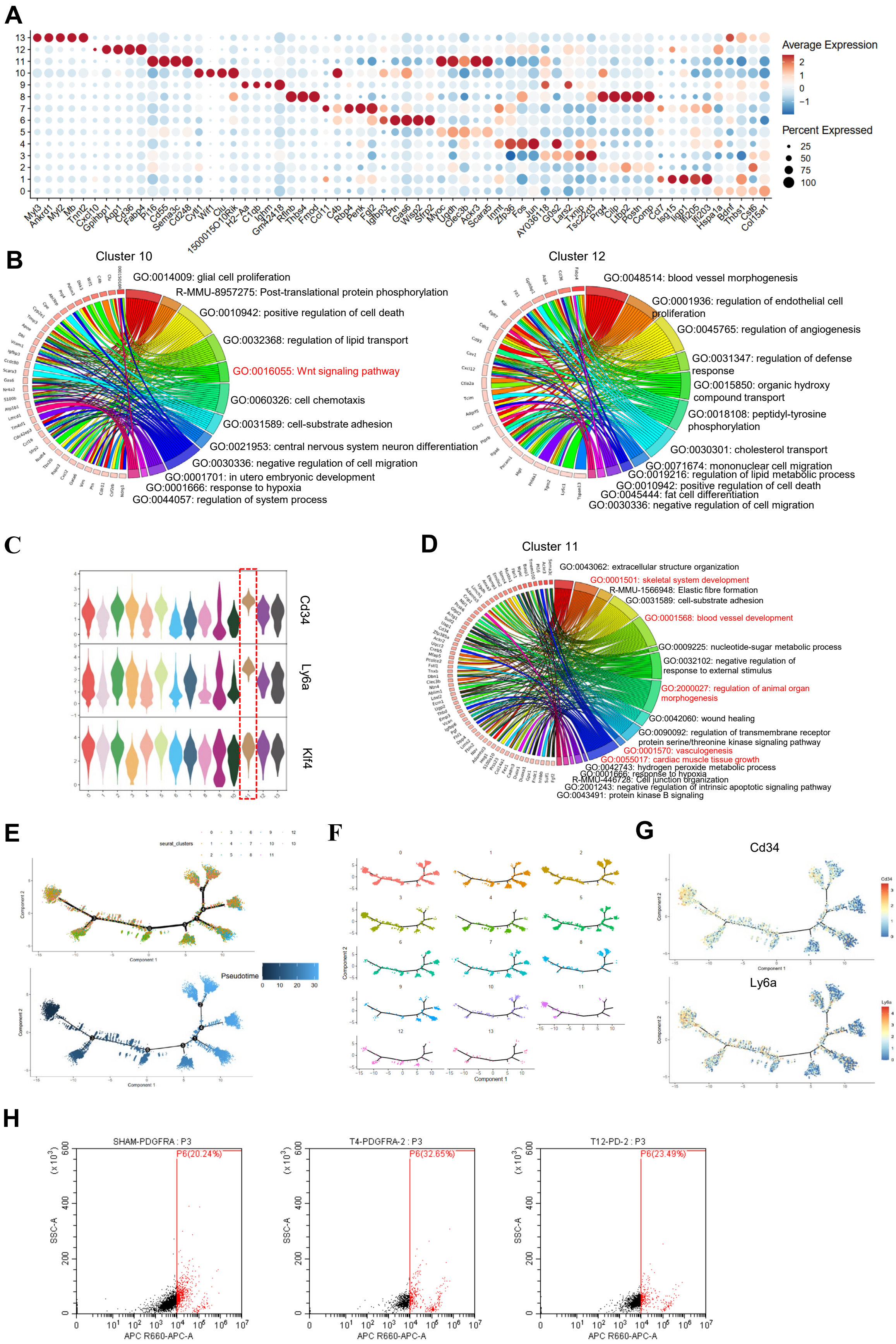

A
Average Expression
Percent Expressed
B
Cluster 10
GO:0014009: glial cell proliferation
R-MMU-8957275: Post-translational protein phosphorylation
GO:0010942: positive regulation of cell death
GO:0032368: regulation of lipid transport
GO:0016055: Wnt signaling pathway
GO:0060326: cell chemotaxis
GO:0031589: cell-substrate adhesion
GO:0021953: central nervous system neuron differentiation
GO:0030336: negative regulation of cell migration
GO:0001701: in utero embryonic development
GO:0001666: response to hypoxia
GO:0044057: regulation of system process
Cluster 12
GO:0048514: blood vessel morphogenesis
GO:0001936: regulation of endothelial cell proliferation
GO:0045765: regulation of angiogenesis
GO:0031347: regulation of defense response
GO:0015850: organic hydroxy compound transport
GO:0018108: peptidyl-tyrosine phosphorylation
GO:0030301: cholesterol transport
GO:0071674: mononuclear cell migration
GO:0019216: regulation of lipid metabolic process
GO:0010942: positive regulation of cell death
GO:0045444: fat cell differentiation
GO:0030336: negative regulation of cell migration
C
Cd34
Ly6a
Klf4
D
Cluster 11
GO:0043062: extracellular structure organization
GO:0001501: skeletal system development
R-MMU-1566948: Elastic fibre formation
GO:0031589: cell-substrate adhesion
GO:0001568: blood vessel development
GO:0009225: nucleotide-sugar metabolic process
GO:0032102: negative regulation of response to external stimulus
GO:2000027: regulation of animal organ morphogenesis
GO:0042060: wound healing
GO:0090092: regulation of transmembrane receptor protein serine/threonine kinase signaling pathway
GO:0001570: vasculogenesis
GO:0055017: cardiac muscle tissue growth
GO:0042743: hydrogen peroxide metabolic process
GO:0001666: response to hypoxia
R-MMU-446728: Cell junction organization
GO:2001243: negative regulation of intrinsic apoptotic signaling pathway
GO:0043491: protein kinase B signaling
E
Pseudotime
Component 1
Component 2
F
Component 1
Component 2
G
Cd34
Ly6a
H
SHAM-PDGFRA : P3
P6(20.24%)
T4-PDGFRA-2 : P3
P6(32.65%)
T12-PD-2 : P3
P6(23.49%)
SSC-A
APC R660-APC-A

Figure S2. Related to Figure 2

(A). Dotplot showing expression of top five genes expressed in FB subclusters. Dot size reflects the percentage of cells expressing the selected gene in each cell cluster. **(B)**. Chord plot showing the GO enrichenment of selected subcluster. **(C)**. Violin plot showing expression of selected spc marker gene in each subclusters. **(D)**. Chord plot showing the GO enrichment biological processes of selected subcluster. **(E)**. Trajectory analysis of each subclusters. **(F)**. Trajectory analysis of each subclusters of fibroblasts. **(G)**. Pseudotime-dependent expression of Cd34 and Ly6a. **(H)**. Flow cytometry analysis of the percentage of PDGFRA+ cell (Fibroblast) in the heart. T0, TAC 0; T4, TAC 4; T12, TAC 12.

scale bar
scale bar

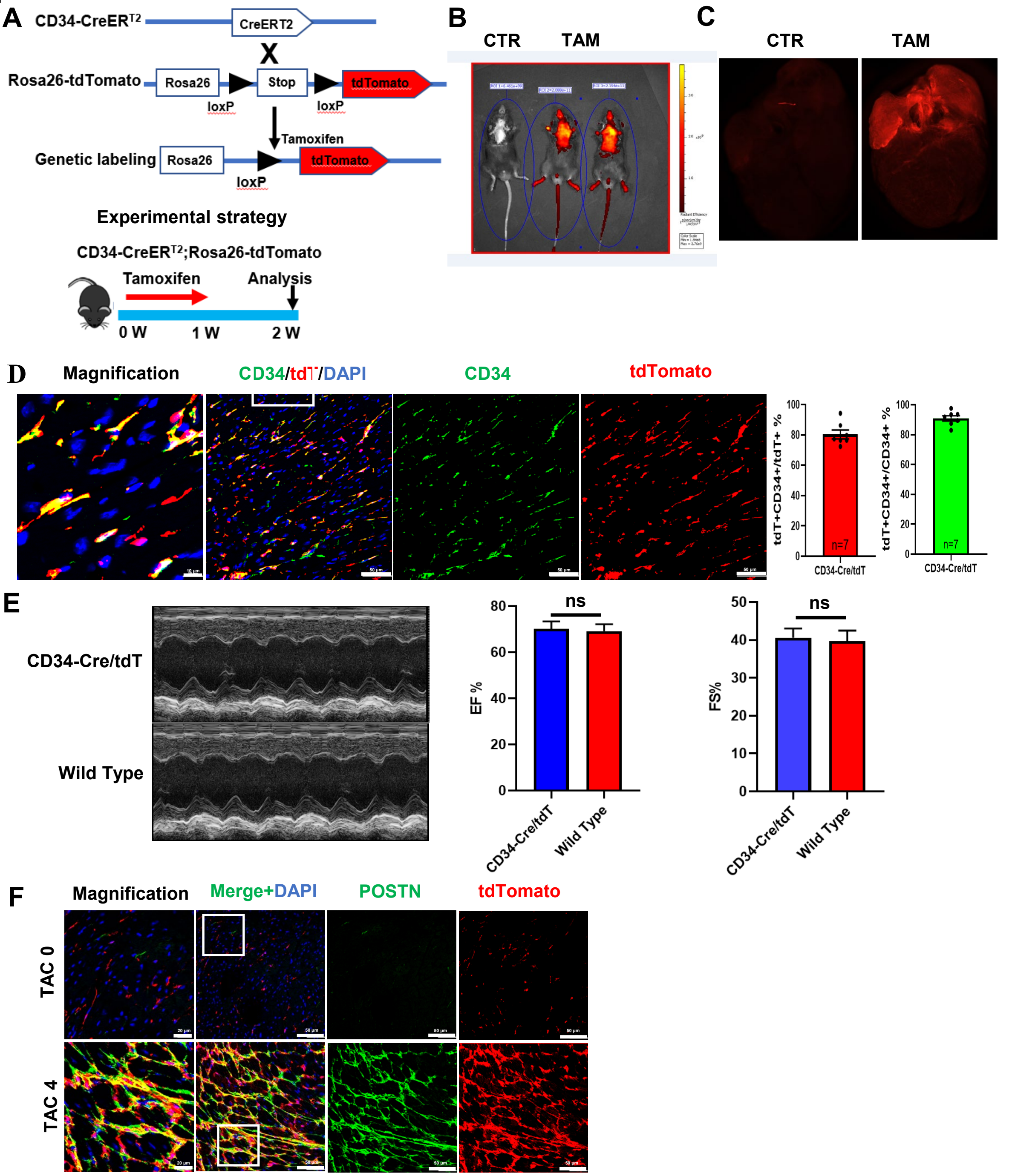

Figure S3. Related to Figure 3

(A). Construction of CD34-CreERT2; Rosa26-tdTomato lineage tracing mouse model; Experimental scheme whereby *Cd34*-CreER^{T2}; R26-tdTomato (Cre/tdT) mice were given tamoxifen for 1 weeks before sham or TAC surgery. **(B)**. In vivo imaging of CD34-CreERT2;Rosa26-tdTomato mice with or without tamoxifen induction. **(C)**. Whole mount staining of heart of CD34-CreERT2;Rosa26-tdTomato mice with or without tamoxifen induction. CTR, mice without tamoxifen induction; TAM, mice with tamoxifen induction. **(D)**. Representative cross-sections of of CD34-CreERT2;Rosa26-tdTomato mice by immunofluorescence staining with tdTomato and CD34. **(E)**. Echocardiographic measurements of left ventricle ejection fraction (EF) and fractional shortening (FS) in in wild-type (WT) and CD34-Cre/tdT (CD34-CreERT2;Rosa26-tdTomato) mice. N=7 per group. Data shown were mean ± SEM, ns: not significant. **(F)**. Representative images showing specific cell identification by staining with tdTomato and POSTN in different groups (sham, TAC 4) by immunofluorescence staining(scale bar= 20 μm or 50 μm).

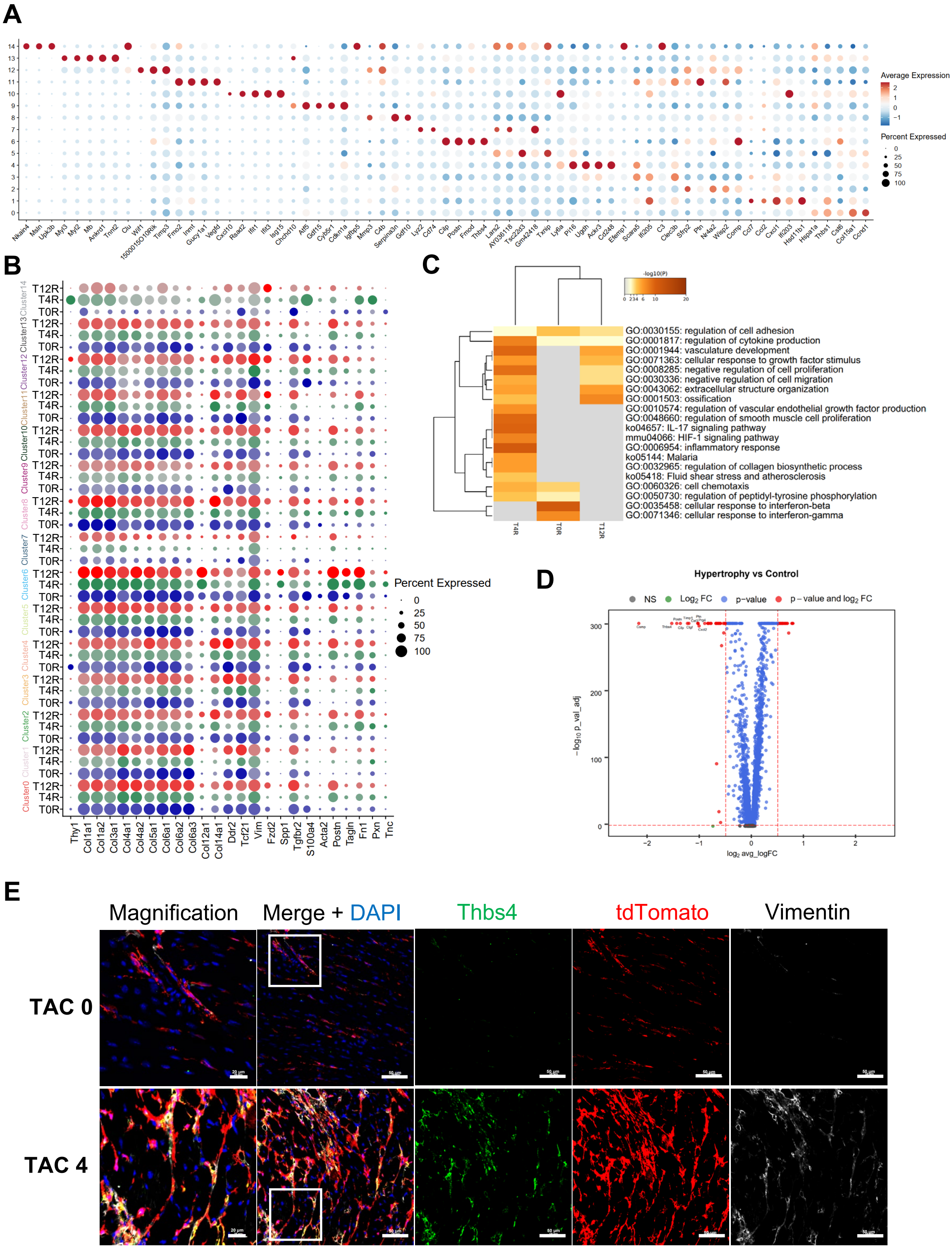

Figure S4. Related to Figure 3

(A). Dotplot showing expression of top five genes expressed in FB subclusters. Dot size reflects the percentage of cells expressing the selected gene in each cell cluster. (**B**). Dotplot showing the expression of genes related to ECM proteins and fibroblast activation at 0, 4 and 12 weeks after TAC surgery. (**C**). Bar plot showing the GO enrichment of different expressed gene between different groups (T0 R, Sham; T4 R, TAC 4R;T12 R, TAC 12R). (**D**). Volcano plot showing different expressed gene between different groups. (P<0.01,log2fc>1 was labeled). (**E**). Representative images of tdTomato and fibroblast markers (Thbs4, Vimentin) by immunofluorescence staining between different groups (TAC 0, TAC 4) (scale bar= 20 μm or 50 μm).

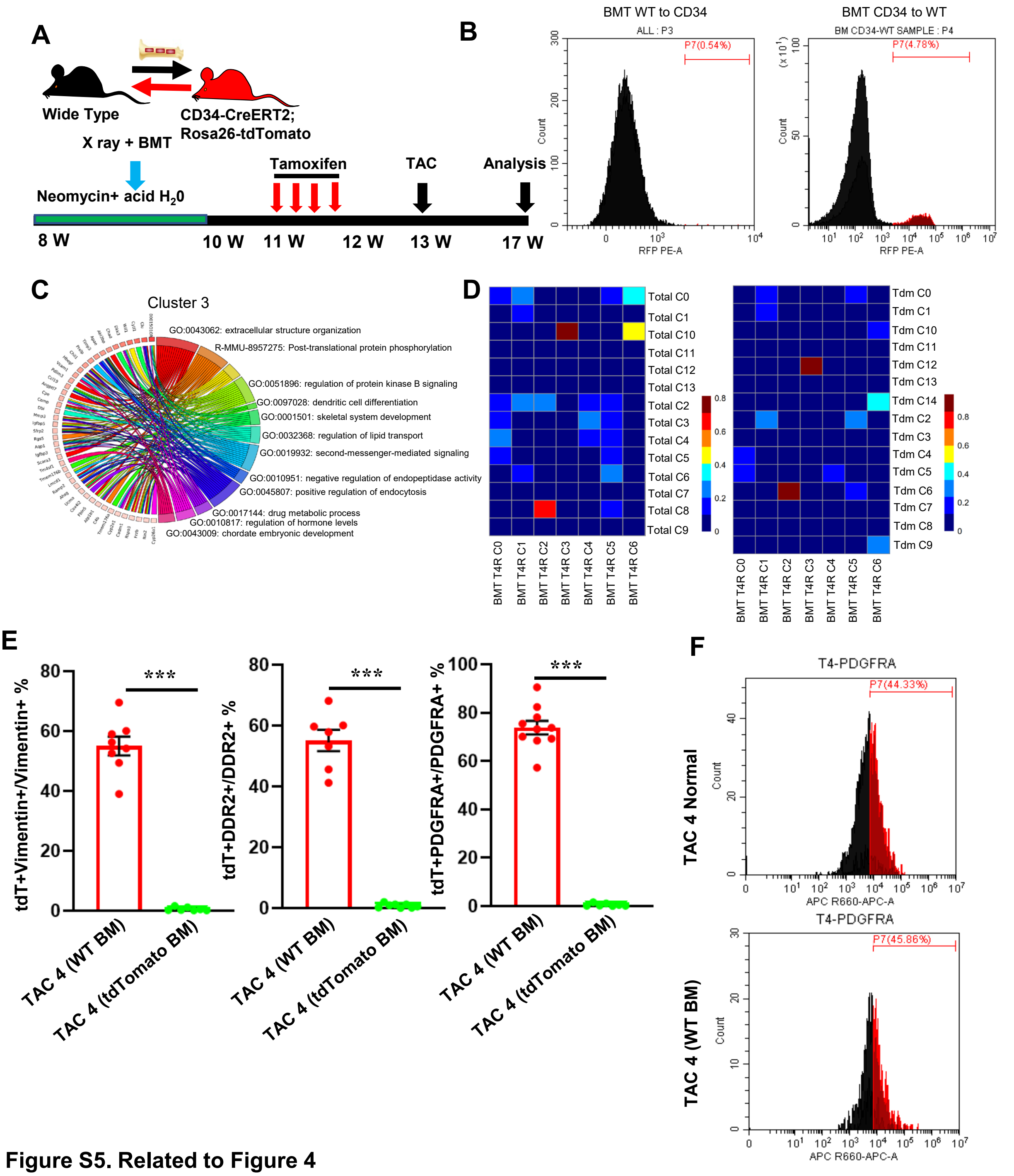

Figure S5. Related to Figure 4

(A). Schematic depicting the pipeline of bone marrow transplantation. Chimeric mice were created by transplanting bone marrow cells from wild-type C57BL/6J mice to CD34-CreERT2;Rosa26-tdTomato (BMT WT TO CD34), or from CD34-CreERT2;Rosa26-tdTomato to WT (BMT CD34 to WT), further treated with tamoxifen and subjected to TAC surgery. (**B**). Representative Flow cytometry analysis was applied to confirm the reconstitution of tdTomato+ bone marrow cells. (**C**). Chord plot showing the GO analysis of cluster3. (**D**). Cluster similarity among different groups. (**E**). Graph showing percentage of tdTomato expression in vimentin+, DDR2+ fibroblast and PDGFRA+ Fibroblast. Data represent mean ± SEM, n=7. *P <0.05; **P < 0.01; ***P < 0.001. **(F)** Representative Flow cytometry analysis showing the percentage of tdTomato^{+}PDGFRA^{+} cell in the heart between two group. Normal: *Cd34*-CreERT2;Rosa26-tdTomato mice without bone marrow transplant; BM, bone marrow; BMT, bone marrow transplantation; WT BM, bone marrow from wild-type C57BL/6J mice; tdTomato BM, bone marrow from CD34-CreERT2;Rosa26-tdTomato.

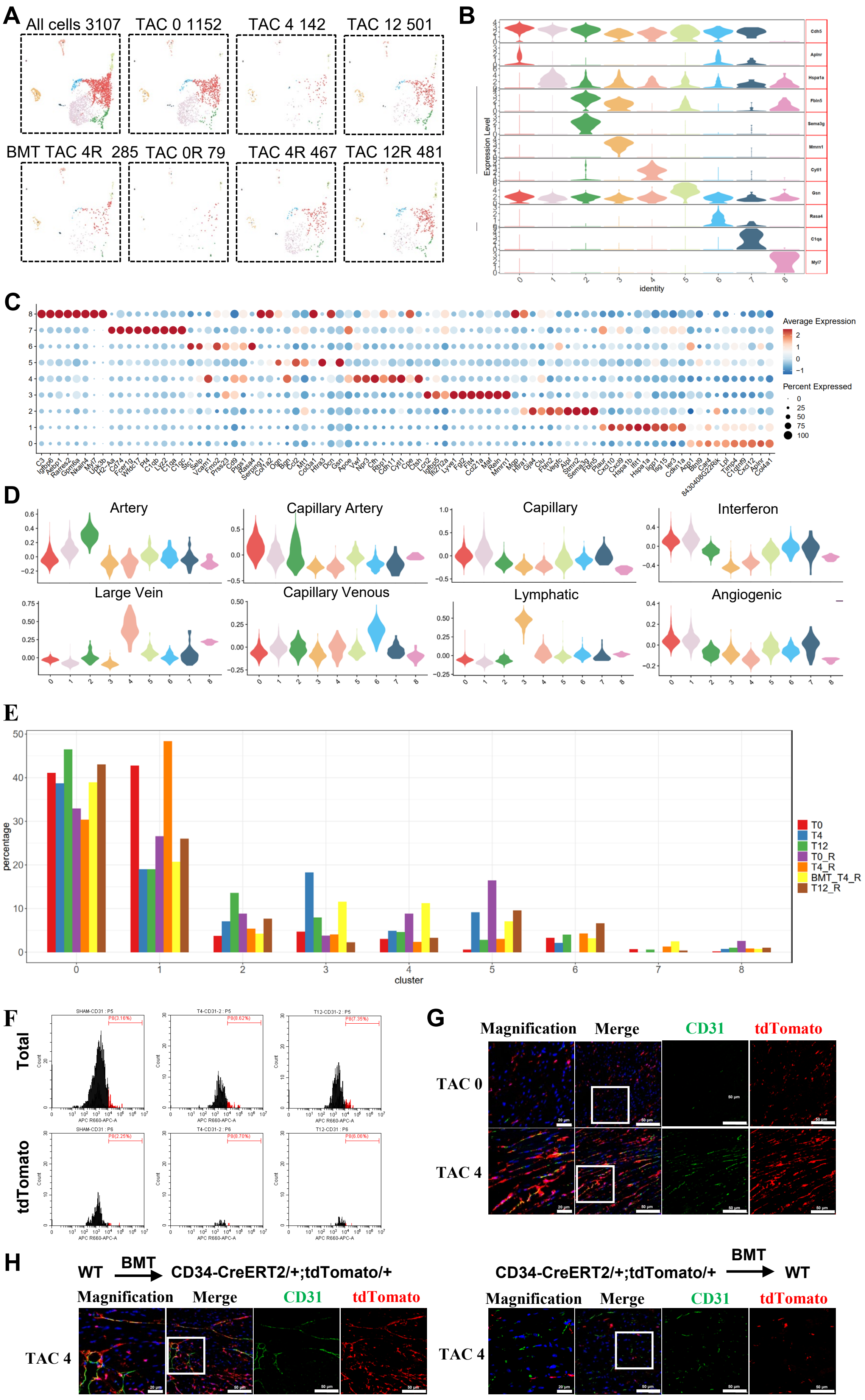

A
All cells 3107
TAC 0 1152
TAC 4 142
TAC 12 501
BMT TAC 4R 285
TAC 0R 79
TAC 4R 467
TAC 12R 481
B
Expression Level
identity
Cdh5
Aplnr
Hspa1a
Fbln5
Sema3g
Mmrn1
Cytl1
Gsn
Rasa4
C1qa
Myl7
C
Average Expression
Percent Expressed
D
Artery
Capillary Artery
Capillary
Interferon
Large Vein
Capillary Venous
Lymphatic
Angiogenic
E
percentage
cluster
T0
T4
T12
T0_R
T4_R
BMT_T4_R
T12_R
F
Total
tdTomato
G
Magnification
Merge
CD31
tdTomato
TAC 0
TAC 4
H
WT
BMT
CD34-CreERT2/+;tdTomato/+
Magnification
Merge
CD31
tdTomato
TAC 4
CD34-CreERT2/+;tdTomato/+
BMT
WT
Magnification
Merge
CD31
tdTomato
TAC 4

Figure S6. Characterization of EC clusters of different stages of pathological cardiac hypertrophy by ScRNA-seq. (A). Umap plot showing the distribution of sub-clusters in ECs of different datasets (resolution 0.2) at 0, 4 and 12 weeks after TAC surgery . n=3107 cells. **(B)**. Violin plot showing the expression of selected marker. **(C)**. Dotplot showing expression levels of top ten differentially expressed genes in each cell cluster. Dot size reflects the percentage of cells expressing the selected gene in each cell cluster. **(D)**. Violin plot showing the expression of selected gene sets among subclusters. **(E)**. Bar chart showing the percentage of sub-clusters in datasets of different groups. **(F)**. Flow cytometry showing the percentage of ECs in different stages of cardiac hypertrophy. (**G)**. Immunofluorescence staining of tdTomato and ECs markers (CD31) in sham and TAC 4 group. **(H)**. Immunofluorescence staining of tdTomato and ECs markers (CD31) in bone marrow transplantation of WT to CD34-CreERT2; Rosa26-tdTomato mice or CD34-CreERT2; Rosa26-tdTomato mice to WT (scale bar= 20 μm or 50 μm). T0, TAC 0; T4, TAC 4; T12, TAC 12.

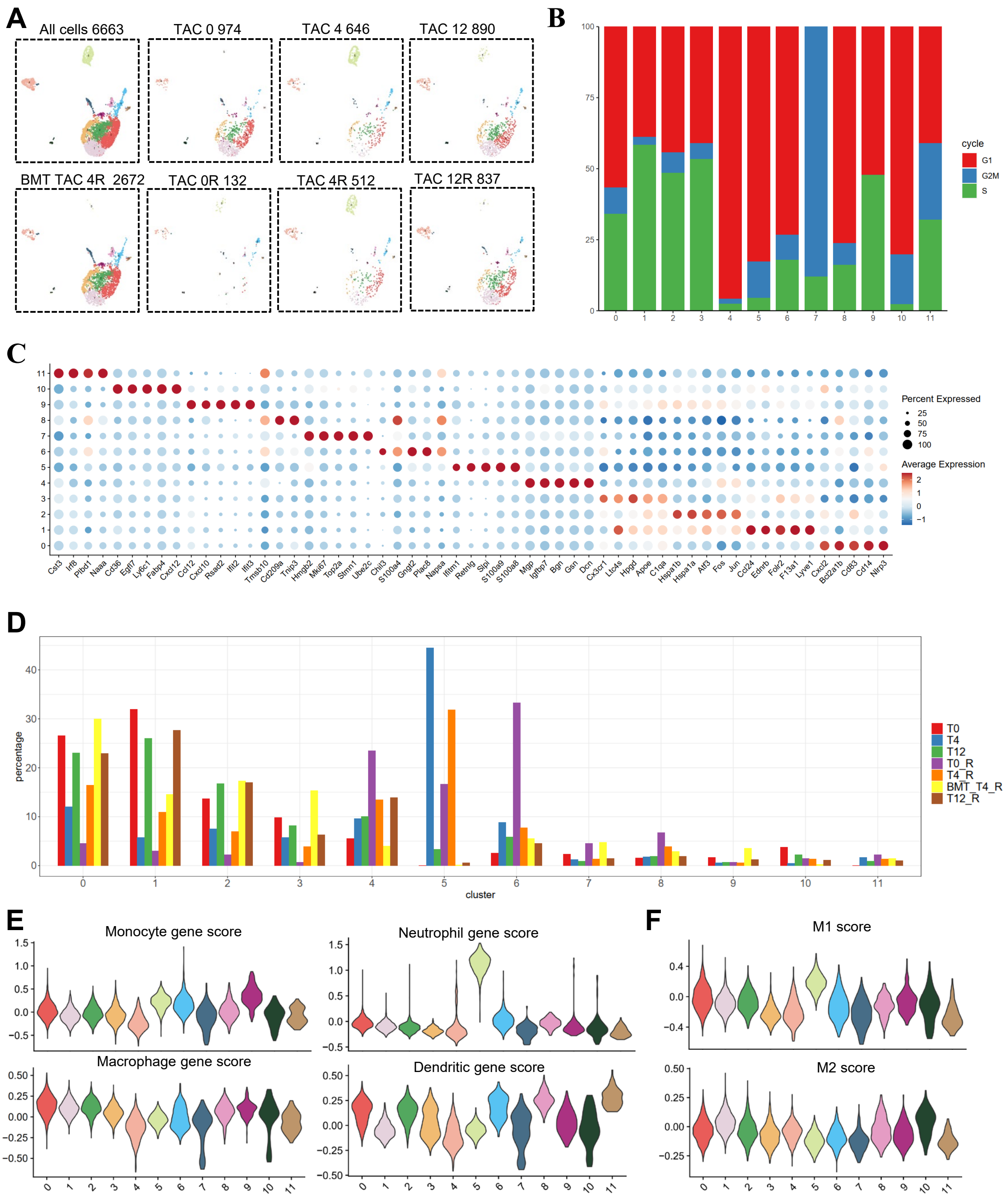

Figure S7. Characterization of MF/Mo/DC clusters of different stages of pathological cardiac hypertrophy by ScRNA-seq. (A). Umap plot showing the distribution of sub-clusters in MF/Mo/DC of different datasets (resolution 0.3) at 0, 4 and 12 weeks after TAC surgery . n=6663 cells. **(B)**. Bar chart showing the percentage cell cycle stages in sub-clusters. **(C)**. Dotplot showing expression levels of top 5 differentially expressed genes in each cell cluster. Dot size reflects the percentage of cells expressing the selected gene in each cell cluster. **(D)**. Bar chart plot showing cell type percentage distribution in each dataset of different groups. T0, TAC 0; T4, TAC 4; T12, TAC 12. **(E)**. Violin plot showing the expression of selected gene sets among subclusters. **(F)**. Violin showing the expression of M1 and M2 genes of cell clusters.

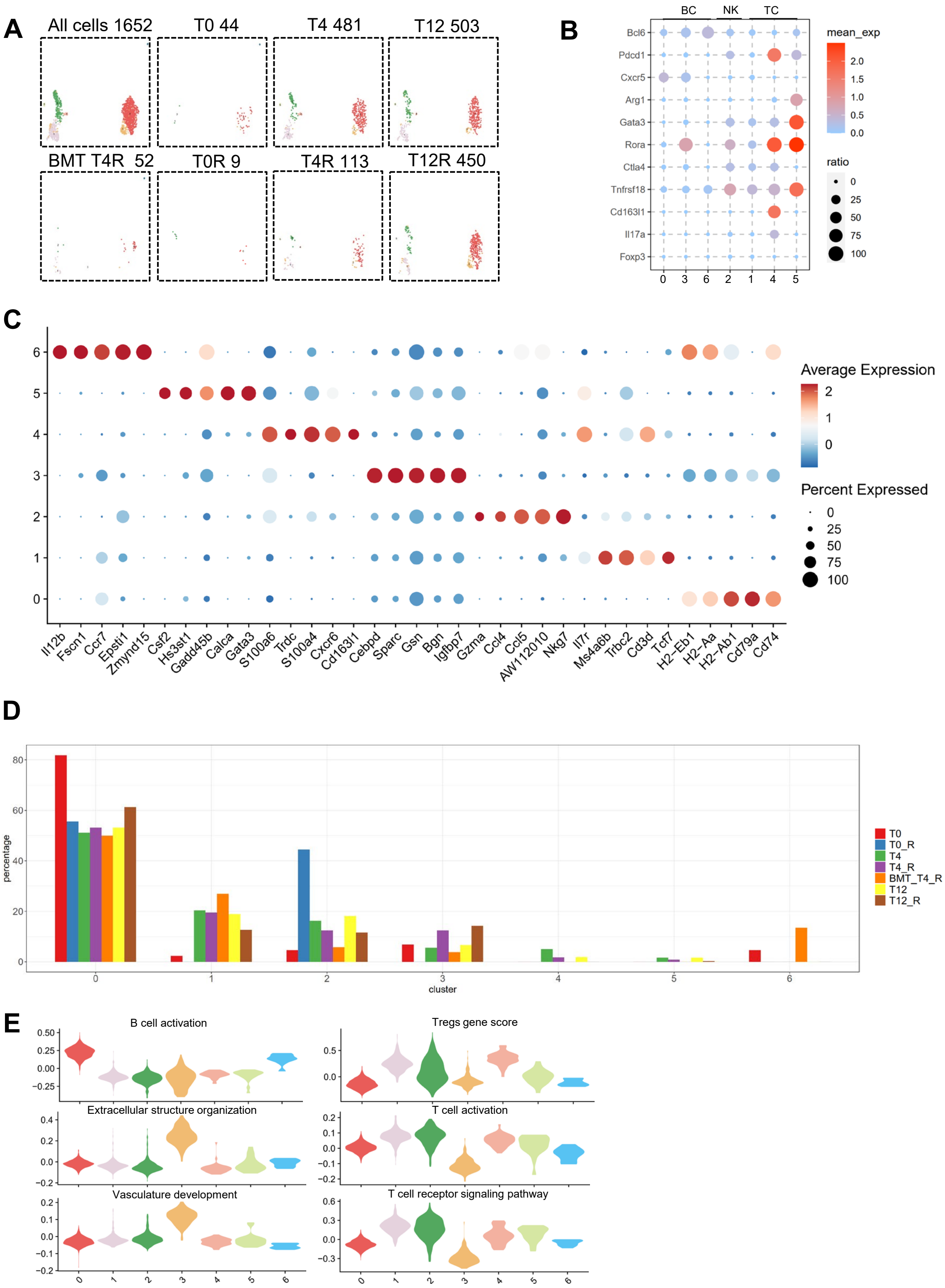

Figure S8. Characterization of Lymphocytes clusters of different stages of pathological cardiac hypertrophy by ScRNA-seq. (A). Umap plot showing the distribution of sub-clusters in Lymphocytes of different datasets at 0, 4 and 12 weeks after TAC surgery . n=1652 cells. **(B).** Dotplot showing expression of selected marker genes in cell clusters. **(C).** Dotplot showing expression levels of top 5 differentially expressed genes in each cell cluster. **(D).** Bar chart plot showing cell type percentage distribution in each dataset of different groups. **(E).** Violin plot showing the expression of selected gene sets among subclusters.

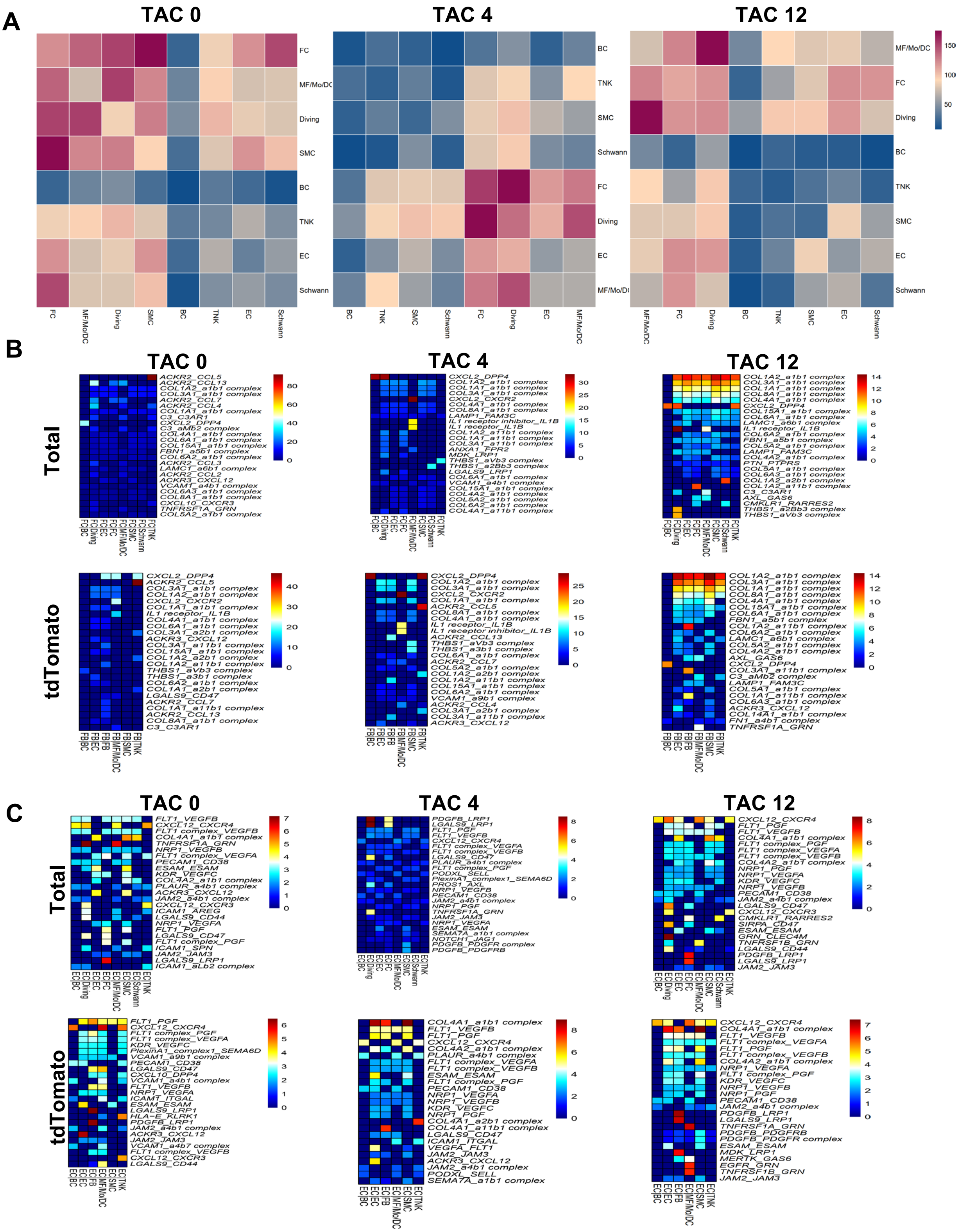

Figure S9. cell communication between different cell types at different stages of pathological cardiac hypertrophy. (A). Heatmap summarizing interconnections between different non-cardiac cell types from hearts at 0, 4 and 12 weeks after TAC surgery. **(B).** Heatmap showing top ligands-receptor pairs by fibroblasts at different stages of cardiac hypertrophy. **(C).** Heatmap showing top ligands-receptor pairs by endothelial cells at different stages of cardiac hypertrophy.

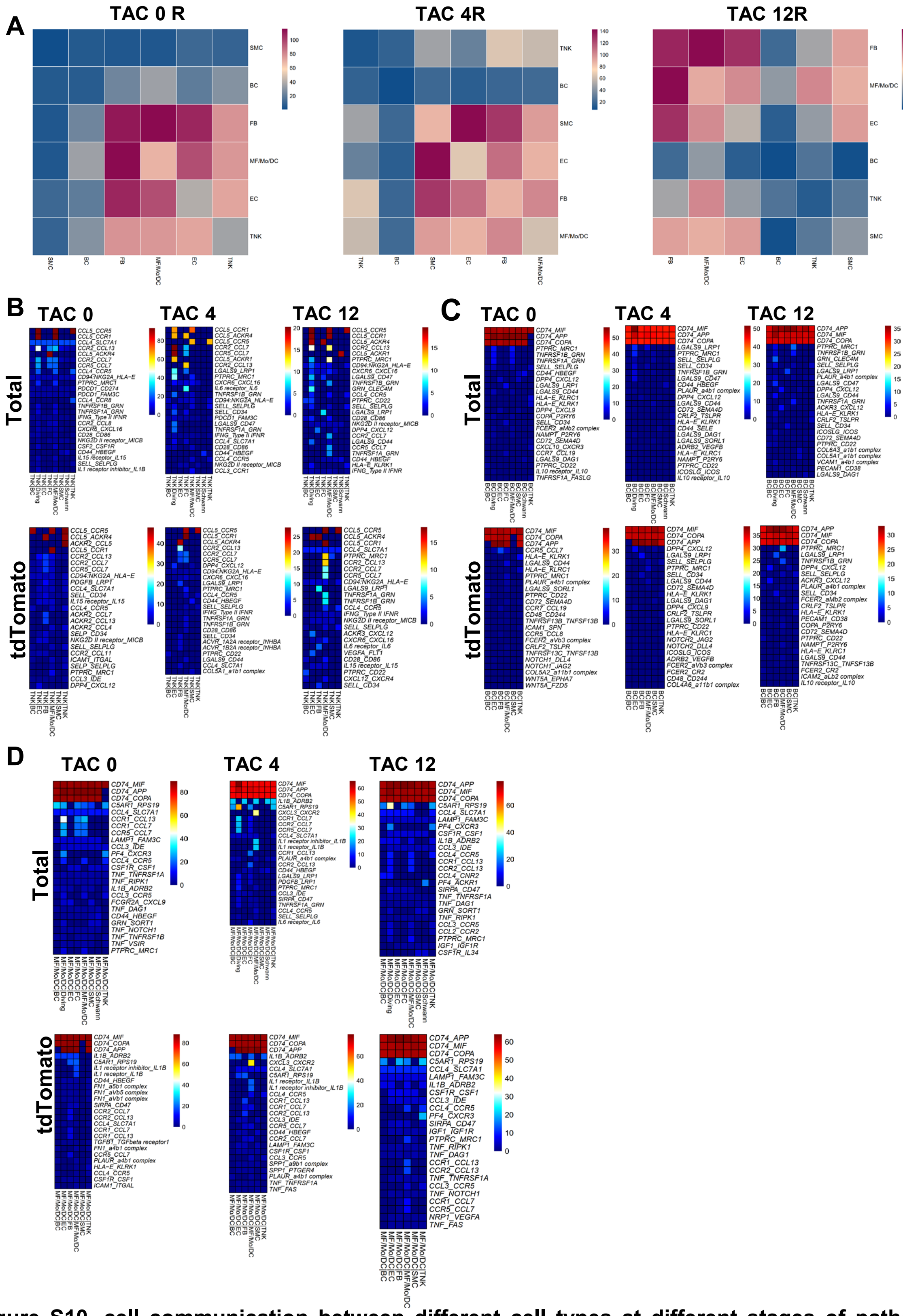

Figure S10. cell communication between different cell types at different stages of pathological cardiac hypertrophy.

(A). Heatmap summarizing interconnections between different cardiac cell types derived from CD34+ cells from hearts at 0, 4 and 12 weeks after TAC surgery. **(B)**. Heatmap showing top ligands-receptors by TB cells at different stages of cardiac hypertrophy. **(C)**. Heatmap showing top ligands-receptors by B cells at different stages of cardiac hypertrophy. **(D)**. Heatmap showing top ligands-receptors by MF/Mo/DC at different stages of cardiac hypertrophy.

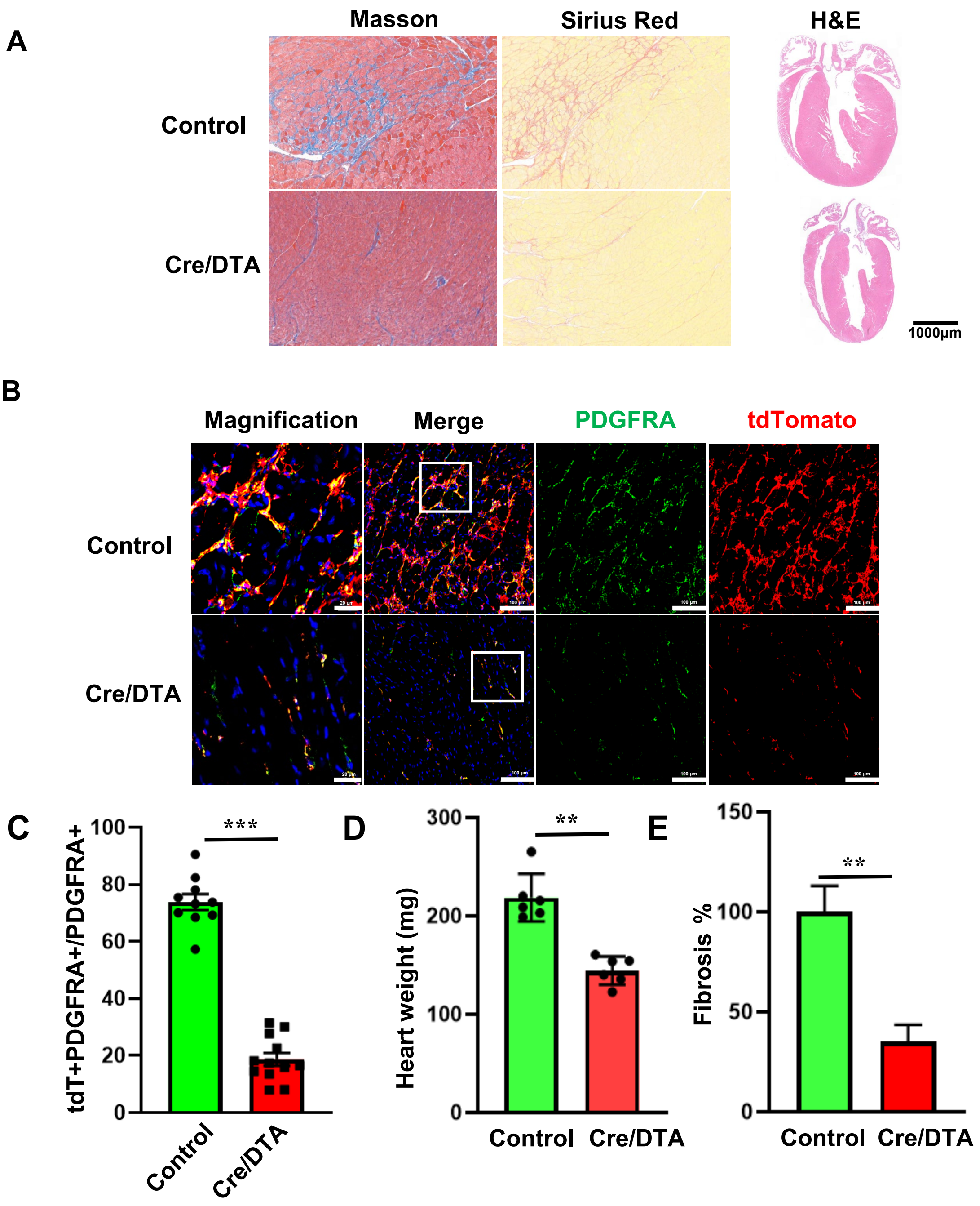

Figure S11. Effect of depletion of CD34+ cells on myocardial fibrosis

(A). Representative morphological images by H&E, Sirius red, Masson staining of hearts from Cre/DTA or control mice at 4 weeks after TAC surgery; Sirius red, Masson staining showing different degrees of fibrosis in control group and Cre/DTA group. **(B)**. Representative immunostaining images showing staining of tdTomato and PDGFRA in control group and Cre/DTA group. n=10 per group. **(C)**. Graph showing percentage of tdTomato expression in PDGFRA+ Fibroblast. Data represent mean ± SEM, n=10. **(D)**. Graph showing the heart weight from Cre/DTA or control mice at 4 weeks after TAC surgery. Data represent mean ± SEM, n=6. **(E)**. Graph showing the fibrosis (Masson staining) in control group and Cre/DTA group. Data represent mean ± SEM, n=6.

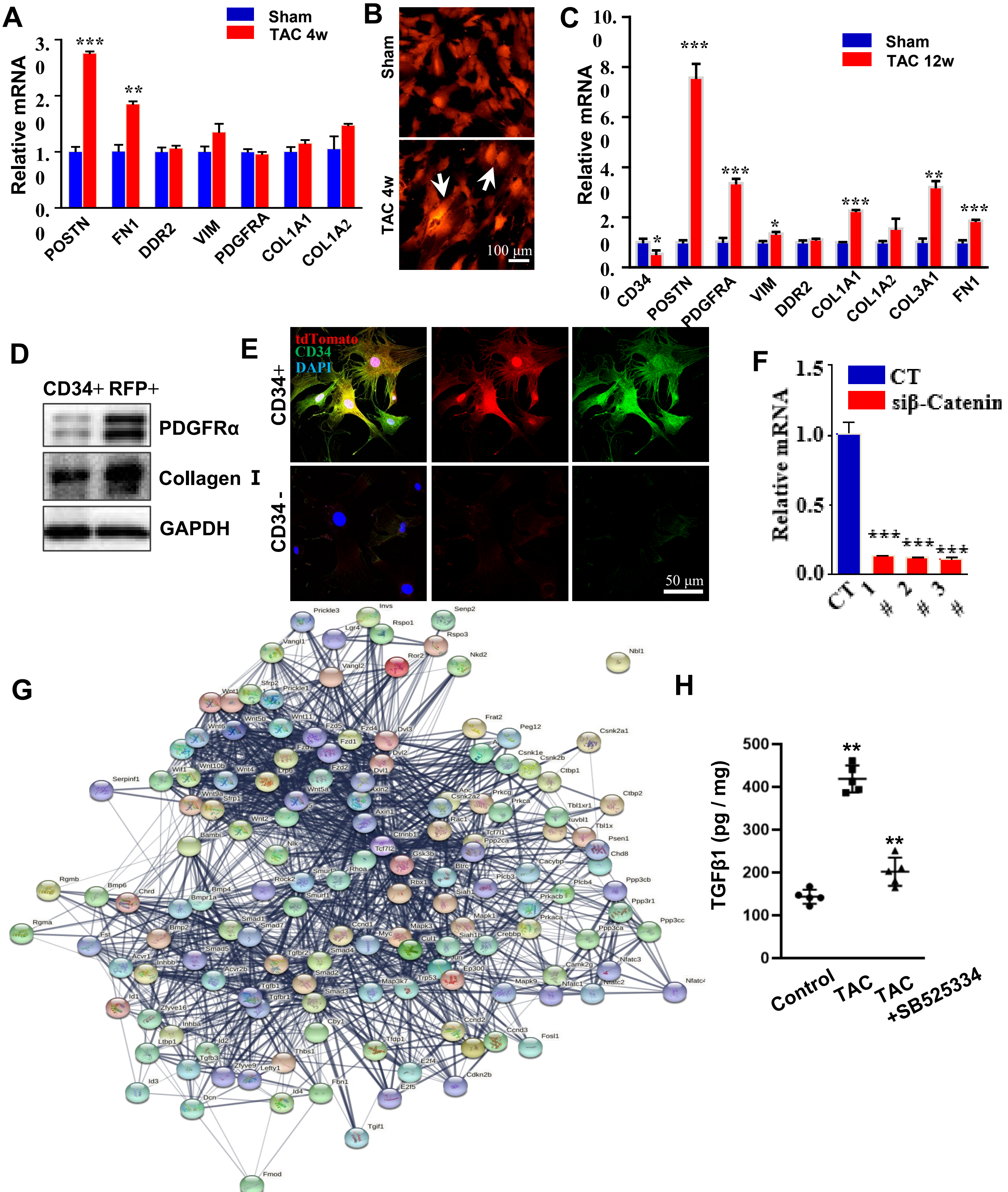

Figure S12. Heart-derived CD34⁺ cells can be directed into fibroblastic cells. **A**. mRNA levels of fibroblastic markers between sham and TAC group, GAPDH was used as internal control, n=6. **B**. cell size and cell morphology of tdTomato between sham and TAC 4W group. **C-D**. mRNA and protein levels of fibroblastic markers between sham and TAC 12W group. **E**. Immunofluorescence staining of CD34 in CD34⁺ cells. **F**. Validation of β-catenin siRNA efficiency. **G**. Interactions of Wnt and TGFβ pathway genes enriched by pseudotime analysis. **H.** Graph showing the level of TGFβ1 in heart tissue by ELISA. Data represent mean ± SEM, n=5. SB525334, a selective TGFβ1 receptor inhibitor.

A

	Control	HF
Estimated Number of Cells	16635	7234
Mean Reads per Cell before Aggregation	66,672	75,415
Median Genes per Cell before Aggregation	2,113	948
Total Genes Detected	27,937	24,640
Number of Cells after filtering	15927	6610

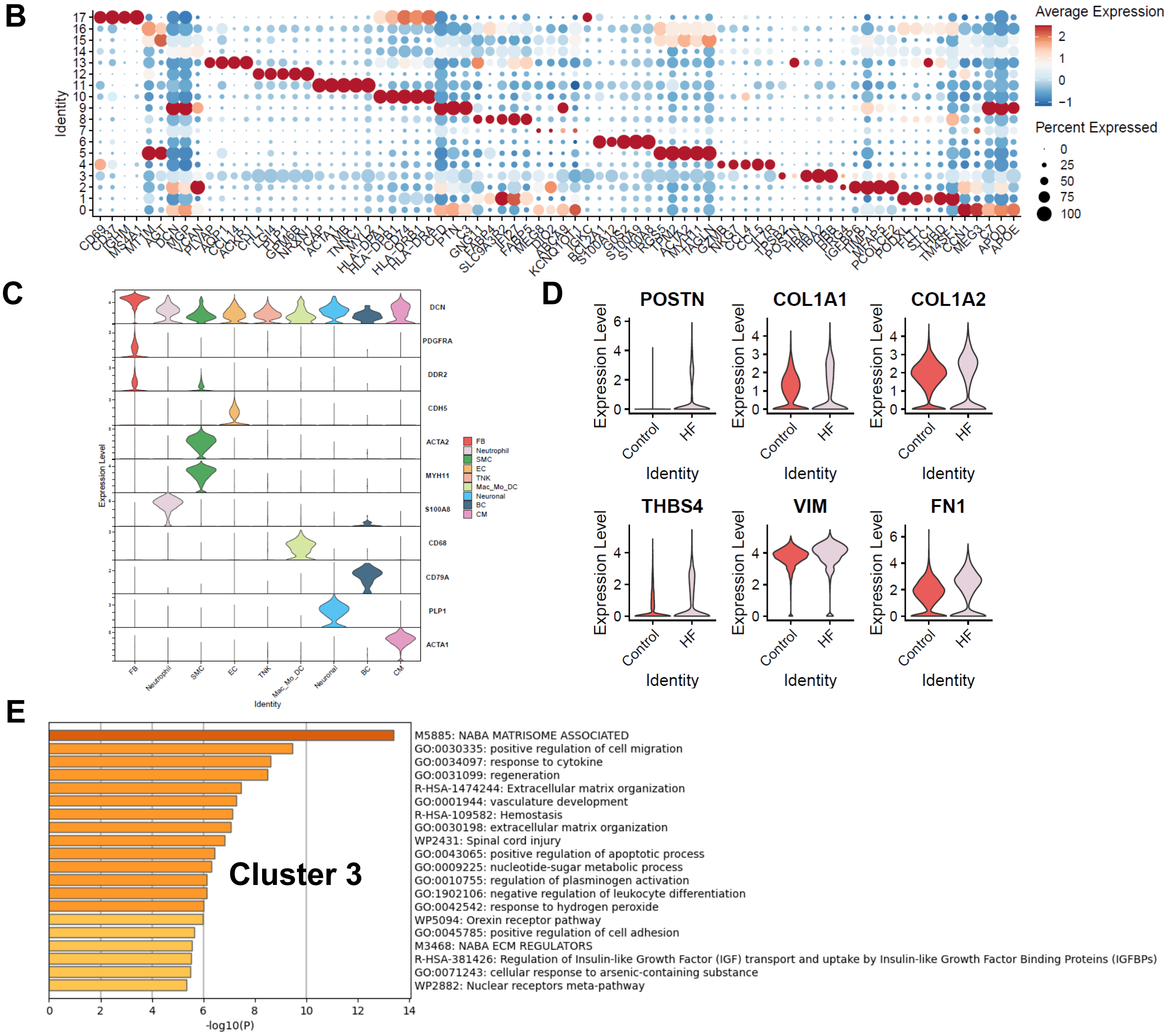

Figure S13. Related to Figure 7.

(A). Basic quality control metrics of indicated datasets before and after cell ranger aggregation and filtering. **(B)**. Dot plot showing expression of top five genes expressed in each subcluster. Dot size reflects the percentage of cells expressing the selected gene in each cell cluster. **(C)**. stack violin plot plot showing the expression of selected cell marker gene to define cell clusters from integrated data. **(D).** Violin plot plot showing the expression of selected cell marker gene between Control and Heart failure group**. (E)**. Bar plot showing the GO datasets score among fibroblast subclusters

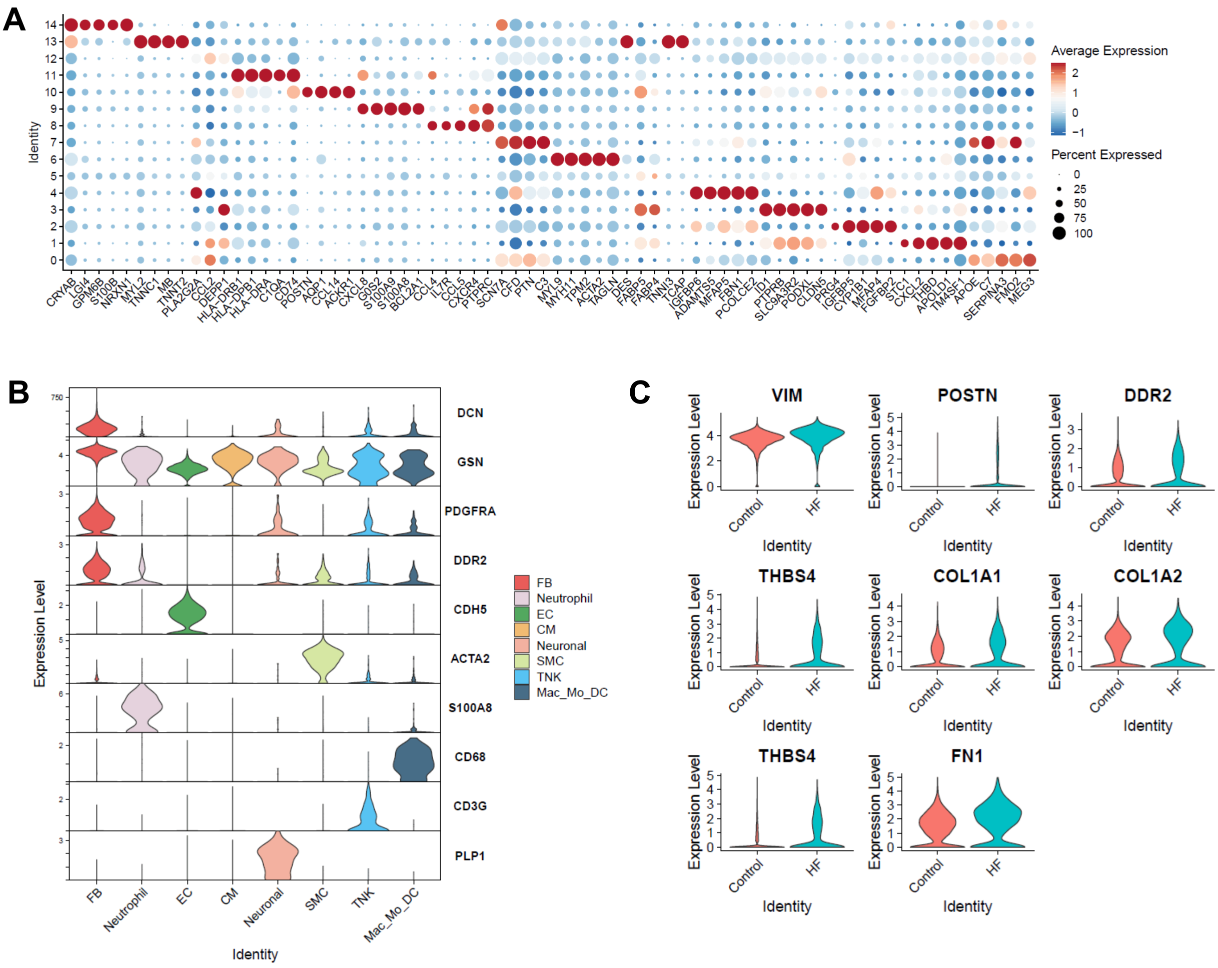

Figure S14. Related to Figure 8. (A). Dot plot showing expression of top five genes expressed of each subcluster in CD34⁺ cells. Dot size reflects the percentage of cells expressing the selected gene in each cell cluster. **(B).** Violin plot plot showing the expression of selected cell marker gene to define cell clusters from integrated data. **(C).** Violin nplot plot showing the expression of selected cell marker gene between Control and Heart failure group.
